# Eye‐Brain Neuroimmune Axis Enables Long‐Term Survival in Glioblastoma by Modulating Brain Immune Surveillance and Neuronal Excitability

**DOI:** 10.1002/advs.76265

**Published:** 2026-06-22

**Authors:** Mingyue Cui, Lulu Qian, Binbin Chu, Xuan Qin, Menglin Wu, Minke Wu, Tongyu He, Baochuan Zhang, Bin Song, Yao He

**Affiliations:** ^1^ Macao Translational Medicine Center Macau University of Science and Technology Taipa Macau SAR China; ^2^ Suzhou Key Laboratory of Nanotechnology and Biomedicine Collaborative Innovation Center of Suzhou Nano Science and Technology (NANO‐CIC) Soochow University Suzhou China

**Keywords:** electrical stimulation, eye–brain neuroimmune axis, glioblastoma, immune activation, neuronal excitability

## Abstract

As an anatomical extension of the central nervous system (CNS), the eye harbors rich neural and immune interfaces with the brain. However, the integrated immunological and neurological nexus between the eye and CNS remains unexplored. Here, we identify the existence of an eye–brain neuroimmune axis by analyzing immune and neuronal responses to eye‐brain electrical stimulation (ES). ES‐assisted intravitreal immunization (IVT) prolonged survival in a murine glioblastoma model, with 33.3% of mice surviving to day 50. Mechanistically, the eye‐brain neuroimmune axis serves two pivotal roles: (1) Immune activation: ES accelerates the rapid drainage of intravitreally delivered antigens to deep cervical lymph nodes (dCLNs), bypassing the BBB and triggering robust CNS‐specific immune responses; (2) Disruption of pathological neuron connectivity: the expression of synaptogenic factors and neuronal excitability can also be mediated under ES treatments. These results uncover an unexplored brain neuroimmune connection, offer new insights into the pathophysiology of eye‐brain diseases and suggest promising avenues for precise diagnostic and therapeutic strategies targeting both ocular and CNS disorders.

## Graphical abstract

The eye–brain neuroimmune axis triggers immune activation and disrupts pathological neuronal connectivity to extend glioblastoma survival.

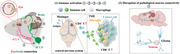

## 1 Introduction

Glioblastoma is recognized as the most aggressive and fatal primary brain tumor in adults, exhibiting particularly poor prognosis in elderly individuals with pronounced immune dysfunction [1, 2, 3, 4, 5]. Consequently, long‐term survival in glioblastoma remains exceedingly rare, with a median overall survival of approximately 15 months and a 5‐year survival rate under 10% even with current standard‐of‐care treatment. Consistently, mice bearing orthotopic GL261 tumors typically exhibit a median survival of only 18 to 25 days, underscoring the severity of the disease in preclinical settings [6]. Historically, the brain was considered an immune‐privileged organ, largely shielded from peripheral immune surveillance by the blood–brain barrier (BBB), which was believed to limit immune cell entry and antigen presentation [7, 8, 9]. However, this paradigm has been challenged by recent discoveries, revealing specialized immunological sites within the CNS, including the meninges, choroid plexus, perivascular spaces, and glymphatic‐lymphatic drainage pathways [10, 11, 12, 13, 14]. These findings challenge the long‐standing concept of CNS immune privilege and suggest that peripheral CNS‐associated structures, such as the eye, may participate in modulating central immune responses.

The eye is anatomically and functionally interconnected with the brain, with the retina functioning as a component of the CNS [15, 16, 17]. This anatomical link extends beyond structure, as the eye and brain also share similarities in immune responses [18, 19]. For example, immune cells in the retina play roles comparable to those in the brain, particularly in the regulation of inflammation and response to injury [20, 21]. Notably, emerging research further indicates a lymphatic connection linking the posterior eye with the brain, enabling a coordinated immune response and emphasizing the existence of eye‐brain immunity [15]. In addition to immunological crosstalk, the eye and brain are also neurologically interconnected through shared developmental origins and direct neural pathways [22, 23]. Extensive research on the brain's nervous system has elucidated mechanisms of neurodegenerative diseases, synaptic plasticity, and neural repair [24, 25]. Meanwhile, ocular neuroscience studies have focused on visual signal processing, survival of retinal ganglion cells and regeneration of the optic nerve, highlighting distinct yet parallel challenges in neuronal resilience and regeneration [26, 27]. Both systems show limited regenerative capacities, with few progenitor cells and non‐replicating nerve cells, making them particularly vulnerable to functional disturbances [22]. This inherent limitation highlights the unique connection between the retina and the brain, allowing the retina not only to function as a sensory organ but also to serve as a crucial window into brain health, enabling non‐invasive monitoring of neurodegenerative diseases [28, 29, 30, 31]. Despite these insights, most studies typically tend to examine the neurological connections separately, rather than understanding their integrated interactions. Consequently, the integrated immunological and neurological nexus between the eye and CNS remains unexplored, underlining the need for further investigation.

**SCHEME 1:**
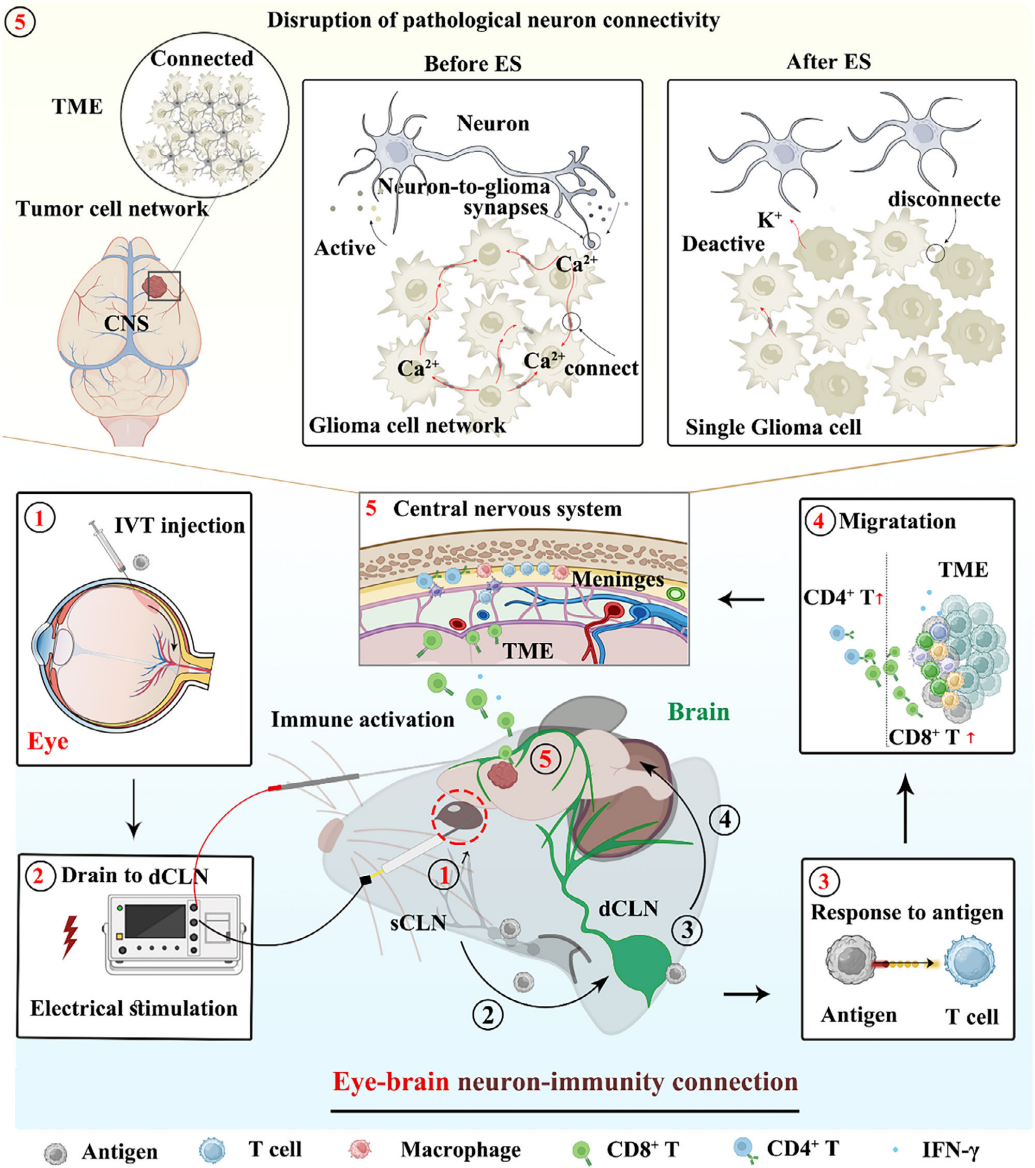
Schematic illustration of eye‐brain neuroimmune connection, highlighting two pivotal roles: (1) immune activation (steps 1 to 5) and (2) disruption of pathological neuron connectivity (step 5).

Here, we provide functional evidence supporting an eye–brain neuroimmune axis that integrates immune activation and neuronal regulation. By analyzing both immune and neuronal responses to the eye‐brain electrical stimulation (ES), we revealed that ES‐assisted intravitreal immunization (IVT) enables long‐term survival in a murine glioblastoma model, with 33.3% of the mice surviving to day 50; whereas anti‐PD1 monotherapy exhibited minimal efficacy, and no mice survived beyond day 32. We further discovered that the eye‐brain neuroimmune axis serves two pivotal roles (Scheme 1). The first function is immune activation, where ES promotes the rapid drainage of intravitreally delivered antigens toward deep cervical lymph nodes (dCLNs), circumventing the BBB and initiating robust CNS‐specific immune responses. This process activates dendritic cells (DCs) in the dCLNs and promotes antigen‐presentation‐associated DC maturation, which is followed by downstream CD4^+^/CD8^+^ T‐cell involvement. The activated T cells subsequently migrate toward the meninges, where they trigger immune responses. Notably, CD4^+^ T cells proliferate and secrete IFN‐γ, which further enhances CD8^+^ T cell activation. The expanded CD8^+^ T cell population then targets tumor cells, effectively suppressing tumor growth and metastasis. The second function involves disruption of pathological neuron–tumor connectivity, as ES modulates synaptogenic gene expression and attenuates neuronal hyperexcitability. Single‐cell RNA sequencing and glioma–neuron co‐culture experiments corroborated these findings, consistent with enhanced immune activation and attenuation of aberrant neuronal excitability. Together, these findings disclose an unexplored eye–brain neuroimmune connection, providing new perspectives on eye‐brain disease mechanisms and paving the way for precise diagnostic approaches for neuroimmune‐related CNS disorders beyond glioblastoma.

## 2 Results and Discussion

### 2.1 Eye‐Brain Circuit Drives Enhanced Lymphatic Drainage and Immune Crosstalk

**FIGURE 1:**
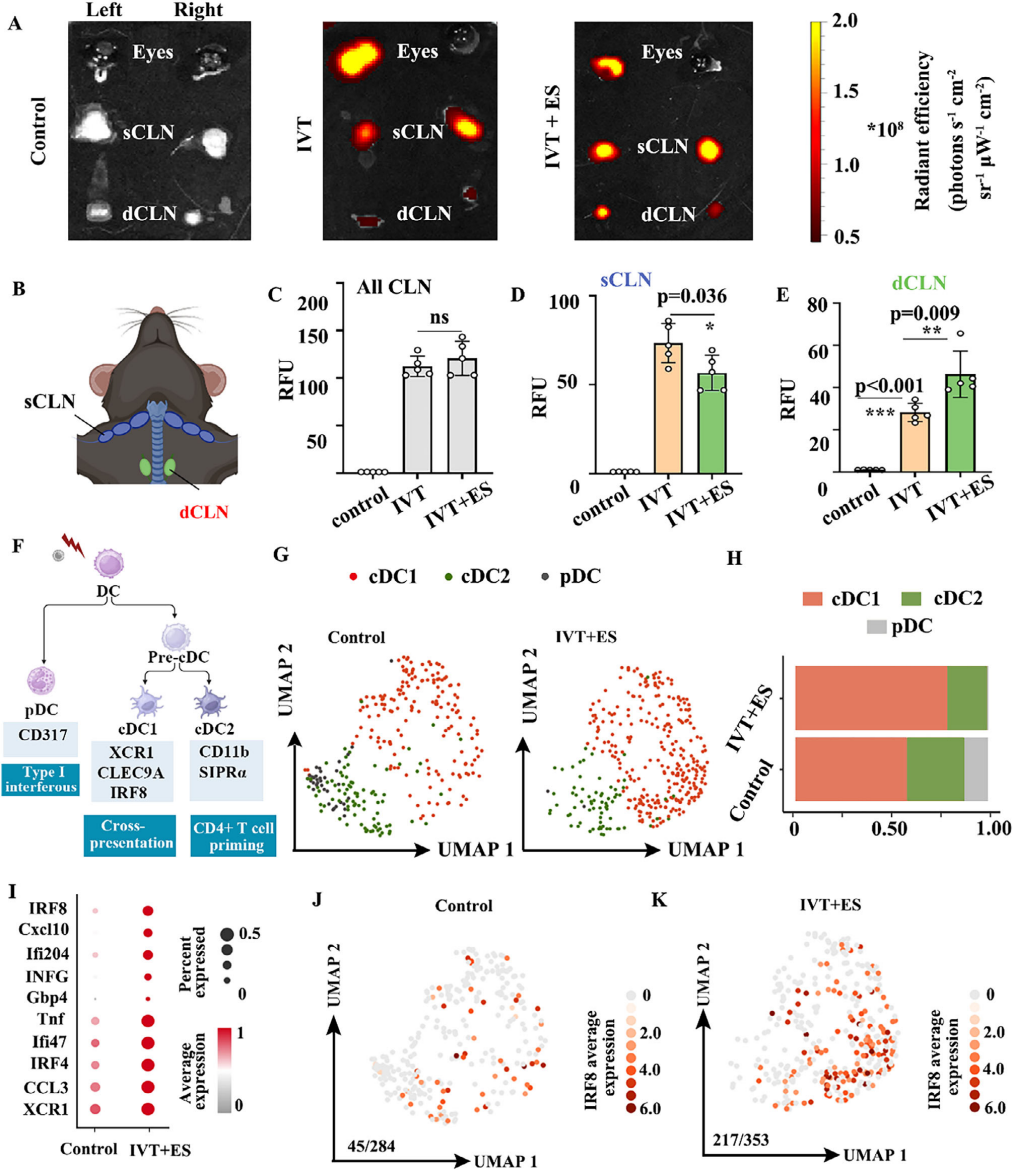
ES enhances the drainage of antigens from the vitreous to dCLNs and promotes DC activation and antigen presentation. (A) Dye was administered into the left eye, and the duration of electrical stimulation (ES) was set to 5 min. The eyes, sCLNs, and dCLNs were harvested and imaged with the IVIS system. Representative images of sCLNs and dCLNs at 1 h post‐injection are presented. (B) Schematic illustration of the anatomical positions of the sCLNs and dCLNs. (C–E) Quantification of distribution across all CLNs, sCLNs, and dCLNs, as measured by IVIS imaging 1 h post‐injection. Relative fluorescence units (RFU) were compared between IVT (*n* = 5) and IVT+ES (*n* = 5) groups. (F) Schematic illustration of DC differentiation into plasmacytoid pDCs, cDC1, and cDC2, highlighting their specific markers and immune functions. (G) UMAP visualizations showing DC subclusters in untreated and IVT+ES groups. (H) Relative proportions of DC subtypes, including cDC1, cDC2, and pDCs, across treatment groups. (I) Relative expression levels of DC activation‐associated markers and cytokine levels produced by cDC1 cells in control and IVT+ES groups. (J, K) UMAP visualizations of IRF8‐cDC1 cells in the dCLNs of untreated and IVT+ES groups. Data are presented as mean ± s.d. Statistical significance was analyzed using one‐way ANOVA followed by Tukey's multiple‐comparisons test. Adjusted *p*‐values for the indicated comparisons are shown in the figure. ^*^p < 0.05, ^**^p < 0.01.

Recent findings have uncovered that the posterior region of the eye drains toward the deep cervical lymph nodes (dCLNs) via lymphatic vessels surrounding the optic nerve sheath, establishing a shared lymphatic network with the central nervous system [15, 32, 33]. In contrast, the effect of ES on the drainage from the vitreous remains unclear, owing to the complexity of the ocular lymphatic vasculature and immune system [34, 35], and is still under investigation. To determine whether ES modulates ocular drainage pathways, fluorescent dextran tracers were introduced into the eye to track transport in both anterior and posterior regions, instead of antigens, via the intravitreal (IVT) route, and monitored dye localization in vivo. We observed that both ES and non‐stimulated dye administered via IVT could be detected in the superficial CLNs (sCLNs) and dCLNs. However, markedly higher dye levels were observed in the dCLNs following ES relative to the non‐stimulated group (Figure 1A and Figure S1). The distribution of sCLNs and dCLNs in mice is depicted in Figure 1b. Quantitative analysis of the relative fluorescence units (RFU) in all lymph nodes was conducted, revealing that in the dCLNs, the RFU in the IVT+ES group was approximately 1.6‐fold greater than that in the IVT group, and about 42.1‐fold greater than the blank group (Figure 1B–E). These results demonstrate that ES of the eye accelerates the drainage of large molecules, such as antigens, from the vitreous to the dCLNs through functional lymphatic vessels surrounding the optic nerve sheath.

To further explore whether ES enhances antigen presentation, thereby strengthening the protective effects of IVT immunization, we harvested the dCLNs and conducted single‐cell RNA sequencing (scRNA‐seq) to characterize dendritic cell (DC) responses. Under stimulation, DCs differentiate into three main subsets: plasmacytoid DCs (pDCs), classical DC1 (cDC1), and classical DC2 (cDC2), each with specific immune functions relevant to antigen presentation [36, 37] (Figure 1F). pDCs, marked by CD317, produce type I interferon (IFN‐I), mediating antiviral responses [38]. cDC1, characterized by *XCR1*, *CLEC9A*, and *IRF8*, are critical for antigen cross‐presentation, enabling extracellular antigens to CD8^+^ T cells through MHC class I molecules, serving as a key driver of antitumor immunity [39]. cDC2, defined by CD11b and SIRPα, primarily activate and regulate naive CD4^+^ T cells, contributing to adaptive immune responses [40]. This analysis aimed to determine how ES affects the abundance and phenotypic characteristics of these DC subsets, particularly in enhancing antigen presentation and immune protection.

We analyzed the DC subclusters according to each well‐defined DC marker (Figure 1G and Figure S2). ES+IVT treatment led to pronounced enrichment of cDC1 cells, reflected by higher proportions and absolute cell numbers. Notably, the cDC1 fraction increased by ∼1.4‐fold compared with untreated mice and accounted for nearly 79% of the total DC compartment. In contrast, the proportion of cDC2 did not change markedly (Figure 1H). Differential gene expression analysis of cDCs revealed upregulation of genes associated with activation and immune responses, including *IRF8, Cxcl10, Ifi204, Ifng, Gbp4, Tnf, Ifi47, IRF4, Ccl3*, and *XCR1*, in cDCs from mice receiving IVT+ES immunization (Figure 1I). Compared to untreated mice, both the proportion and absolute number of IRF8‐cDC1 cells were significantly elevated (Figure 1J,K). These findings indicate that IVT+ES immunization enhances the abundance and activation of cDC1 cells, particularly IRF8‐cDC1, through upregulation of genes associated with antigen presentation and immune responses. This process promotes CD8^+^ T cell activation and enhances immune protection via improved cross‐antigen presentation in dCLNs.

To further test whether the posterior ocular–dCLN pathway is functionally required for ES‐enhanced immune activation, we performed dCLN blockade prior to IVT administration and ES treatment. Tracer‐based imaging showed that dCLN blockade substantially reduced ES‐enhanced tracer accumulation in dCLNs, indicating that efficient ocular antigen transport depends on an intact posterior ocular–dCLN route (Figure S3). Consistently, flow‐cytometric analysis of dCLNs showed that IVT+ES increased the proportion of CD80^+^CD86^+^ mature DCs within the CD11c^+^MHC‐II^+^ population, whereas this increase was markedly attenuated after dCLN blockade (Figure S4). These results functionally support the involvement of the posterior ocular–dCLN pathway in ES‐enhanced antigen transport and DC maturation.

### 2.2 Eye–Brain Neuroimmune Axis Mediates the Central Nervous System (CNS) Specific Immune Protection

Based on the enhanced posterior ocular drainage and the early dCLN DC maturation observed at 24 h, we next examined whether these events were followed by downstream T‐cell‐associated immune responses (Figure S5). Because dCLNs function as brain‐draining lymph nodes and key sites for lymphocyte priming against CNS‐associated antigens, we hypothesized that IVT+ES treatment could promote downstream T‐cell involvement through the eye–brain neuroimmune axis. To test our hypothesis, we first performed transcriptome sequencing (RNA‐seq) analysis of brain tumors from six mice (3 per group) on day 7 post‐treatment (Figures S6–S10). This analysis revealed 175 upregulated and 87 downregulated genes. KEGG pathway and GO enrichment analyses indicated significant upregulation of crucial immune pathways in IVT+ES‐treated mice compared to the untreated group, highlighting enhanced immune activation.

**FIGURE 2:**
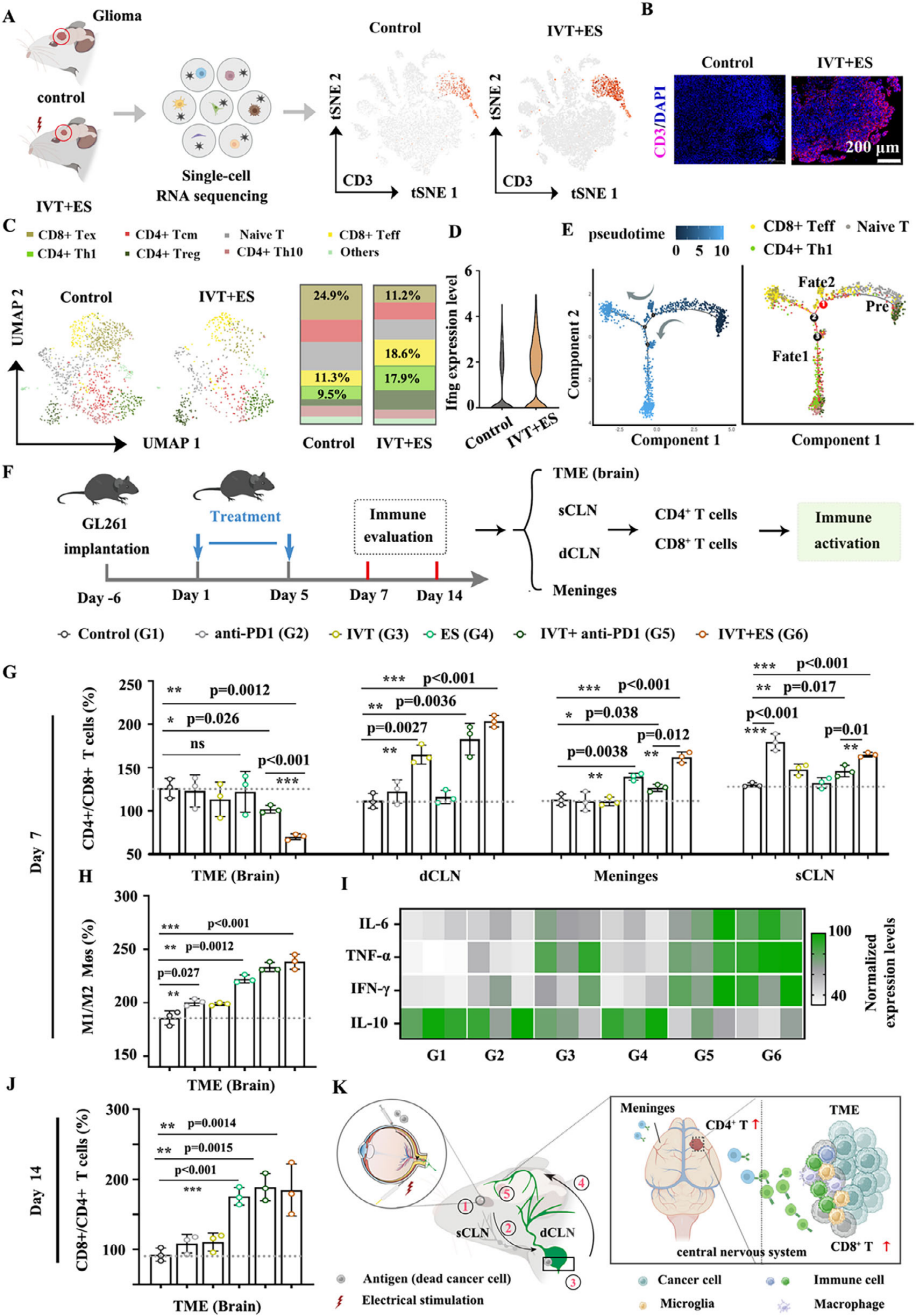
ES combined with IVT immunization effectively activates eye‐brain neuroimmune axis‐mediated CNS‐specific immune responses. (A) Flowchart of glioma single‐cell transcriptomic sequencing combined with t‐SNE mapping of CD3^+^ cells in glioma. (B) Immunofluorescence staining images of CD3^+^ cells in glioma. Scale bars, 200 µm. (C) UMAP visualizations of various T cell populations in the untreated control and IVT+ES groups. Relative distributions of T cell populations, including naive T cells, exhausted CD8^+^ T (CD8^+^ Tex) cells, cytotoxic CD8^+^ T (CD8^+^ Teff) cells, central memory CD4^+^ T (CD4^+^ Tcm), CD4^+^ Th1 cells, CD4^+^ Th10 cells, and regulatory CD4^+^ T cells (CD4^+^ Treg). (D) IFN‐γ expression in T cells in the control and IVT+ES groups. (E) Pseudotime trajectory analysis for T cells, showing the progression of naive T cells into CD8^+^ Teff cells and CD4^+^ Th1 cells. (F) Experimental design used to evaluate in vivo immune responses triggered by different treatments. (G) Quantitative data showing CD4^+^/CD8^+^ T cells in TME, dCLNs, and meninges, and sCLNs induced by different treatments in vivo on day 7 (*n*  =  3 mice). (H) Flow cytometry–based quantification of the M1/M2 macrophage ratio in the TME at day 7 after administration of different formulations (*n* = 3 mice). (I) Heatmap of cytokines secreted in brain TME (*n* = 3). (J) Statistical data illustrating CD8^+^/CD4^+^ T cells in TME by different formulations on day 14 (*n* = 3). (K) Schematic illustration depicting the mechanism through which ES combined with IVT immunization activates the eye–brain axis to induce a CNS‐specific immune response. The proposed model outlines the key events: (1) IVT+ES stimulation; (2) antigen drainage to dCLNs; (3) antigen‐presentation‐associated DC activation and downstream T‐cell involvement; (4) effector T cells migration and functional specialization; (5) amplified immune response leading to tumor cell elimination in the TME. In G–J, data are presented as mean ± s.e.m. All replicates represent biological samples. Statistical significance was analyzed using one‐way ANOVA followed by Tukey's multiple‐comparisons test. Adjusted *p*‐values for the indicated comparisons are shown in the figure. ^*^ *p* < 0.05, ^**^ *p* < 0.01, ^***^ *p* < 0.001.

To further confirm the contribution of T cells to antitumor immune responses triggered by IVT+ES, we conducted scRNA‐seq to examine immune cell alterations within the tumor microenvironment (TME) [41, 42, 43]. The proportion of T cells (*CD3*) was higher in mice treated with IVT+ES than in the control mice, as corroborated by immunofluorescence staining (Figure 2A,B). The T cells population was further classified into several subsets based on well‐defined T cell markers [44, 45] (Figure 2C and Figure S11), mainly including naive T cells (*Tcf7*), exhausted CD8^+^ T cells (CD8^+^ Tex) (*Cd8a, Tim3*), cytotoxic CD8^+^ T cells (CD8^+^ Teff) (*Cd8a*, *Gzmk*), CD4^+^ Th1 cells (*Cd4*, *Ifng*), CD4^+^ Th10 cells (*Cd4*, *IL10*) and regulatory CD4^+^ T (CD4^+^ Treg) cells (*Foxp3*). The fraction of CD8^+^ Teff cells was substantially elevated in the treated group, representing 18.6% of the total CD3^+^ T cell population, whereas the proportion of CD8^+^ Tex cells markedly declined to 11.2% compared to 24.9% in the control group. Similarly, the proportion of CD4^+^ Th1 cells was significantly elevated to 17.9%, approximately twice that of the untreated group (9.5%). CD4^+^ Th1 cells secrete *IFN‐γ*, which enhances CD8^+^ T cell proliferation and cytotoxicity, reshapes the tumor microenvironment, and amplifies antitumor immune responses [46, 47]. The increase in CD8^+^ Teff cells and reduction in CD8^+^ Tex cells improve antigen recognition and alleviate immunosuppressive signaling, effectively activating tumor‐specific immune responses and contributing to tumor suppression. Consistent with these observations, the expression level of *Ifng* was significantly increased, further supporting the enhanced immune activation mediated by the treatment (Figure 2D). Furthermore, pseudotime trajectory analysis of the scRNA‐seq data indicated that the fraction of CD8^+^ Teff cells increased along the trajectory in mice receiving IVT+ES immunization (Figure 2E and Figure S12).

To validate these findings in vivo, we systematically investigated the immune activation effects of IVT+ES in vivo. GL261 tumor‐bearing mice were randomly allocated to 6 groups. On day 7 or 14 post‐treatment, the mice were euthanized, and samples of sCLNs, dCLNs, meninges, and tumor tissues were collected for flow cytometry analysis of immune cells. As relative proportions are more indicative of immune microenvironment alterations than absolute numbers, CD4^+^/CD8^+^ T cell ratios were used to evaluate T cell infiltration [48, 49, 50]. On day 7, T cell analysis in the TME demonstrated that the CD4^+^/CD8^+^ T cell ratios in the IVT+anti‐PD1 and IVT+ES groups were both less than 1, with the IVT+ES group showing an even lower ratio. Notably, CD8^+^ T cells were significantly increased in the IVT+ES group compared to CD4^+^ T cells and other groups (Figures S13 and S14), suggesting enhanced cytotoxic T cell responses in the TME. In the dCLNs and meninges, the CD4^+^/CD8^+^ T cell ratio was significantly higher in the IVT+ES group compared with all other groups, driven by a marked increase in CD4^+^ T cell proportions (Figures S15 and S16). These results indicate that IVT+ES treatment enhances helper T cell responses, potentially amplifying adaptive immune activation in eye‐brain‐draining lymph nodes and meningeal lymphatic vessels. Conversely, in the sCLNs, the CD4^+^/CD8^+^ T cell ratio was highest in the anti‐PD1 group, likely due to the systemic immune effects of peritoneally injected anti‐PD1, which promoted CD4^+^ T cell proliferation and activation (Figure 2G and Figure S17). Additionally, IVT+ES treatment significantly increased the proportion of M1 macrophages, contributing to the reshaping of the TME (Figure 2H and Figures S18 and S19). This was associated with a pronounced increase in *IFN‐γ* cytokine levels within the TME, consistent with the sequencing results, further demonstrating the robust immune activation induced by the treatment (Figure 2I). On day 14, the proportion of CD8^+^ T cells increased in all groups, with a more pronounced elevation observed in the ES, IVT+anti‐PD1, and IVT+ES groups. Similar to the trend recorded on day 7, the proportion of CD4^+^ T cells within the dCLNs and meninges increased on day 14, indicating sustained immune activation (Figures S20 and S21).

Based on these experimental findings, we conclude that ES combined with IVT immunization robustly activates eye‐brain axis‐mediated CNS‐specific immune responses, thereby enhancing tumor elimination through immune mechanisms (Figure 2K). ES facilitates the rapid drainage of antigens from the vitreous to the dCLNs (Step 2), where DCs acquire an antigen‐presentation‐associated mature phenotype, followed by downstream CD4^+^/CD8^+^ T‐cell involvement in dCLNs and brain‐associated immune compartments (Step 3). The activated T cells and antigens then migrate toward the meninges, where they initiate immune responses within the meningeal lymphatic vessels and generate a robust population of CD4^+^ T cells (Step 4). These CD4^+^ T cells secrete *IFN‐γ*, which modulates the immune microenvironment and further enhances the activation and expansion of CD8^+^ T cells within the TME. The expanded CD8^+^ T cell population directly attacks tumor cells by releasing perforin and granzyme B, while also secreting *IFN‐γ* to amplify local immune responses, collectively suppressing tumor growth and metastasis (Step 5). These findings highlight the potential of ES combined with antigen‐based immunization as a precise, non‐invasive strategy for brain tumor immunotherapy.

### 2.3 Downregulation of Neuron‐To‐Glioma Synaptogenic Factor Expression

**FIGURE 3:**
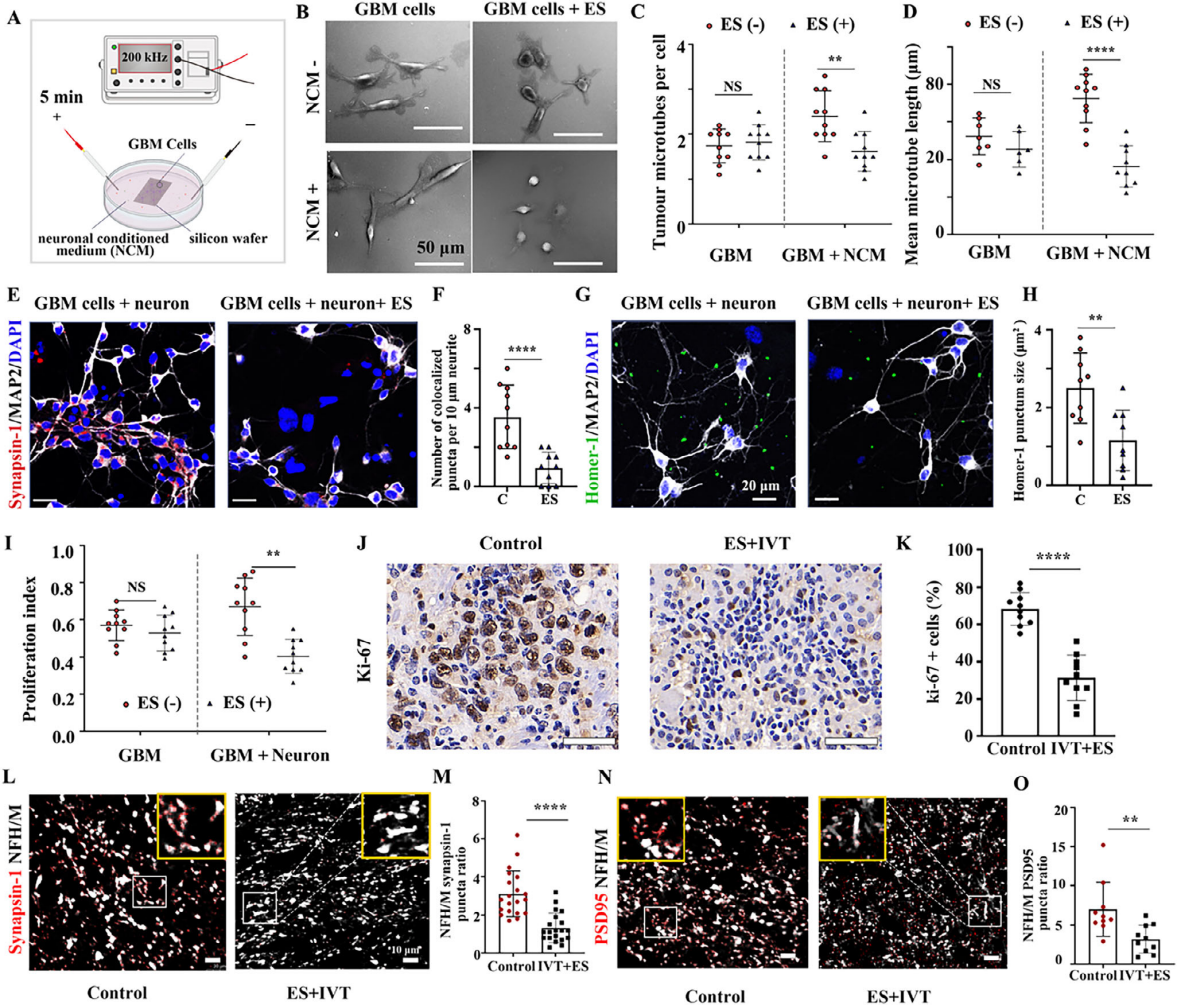
Electrical stimulation suppresses the expression of synaptogenic factors involved in neuron‐to‐glioma synapse formation. (A) Schematic illustration of the experimental setup showing glioblastoma (GBM: GL261) cells exposed to ES in the presence of neuron‐conditioned medium (NCM) on a silicon wafer platform. (B) SEM images of glioma cells cultured with or without NCM under ES or non‐ES conditions, revealing tumor microtubes (TMTs) connecting neighboring cells via cytoplasmic extensions. Scale bars: 50 µm. (C) Quantification of the number of TMTs per cell of b. *n*  =  10. (D) Quantification of average microtube length per cell of b. *n*  =  7 (GBM), *n*  =  6 (GBM+ES), *n*  =  10 (GBM+NCM) and *n*  =  9 (GBM+ NCM+ES). (E, F) Confocal images (e) together with quantification (f) of colocalized presynaptic puncta in neuron–glioma co‐cultures. Red, synapsin‐1; white, MAP2 (neurons); blue, 4′,6‐diamidino‐2‐phenylindole dihydrochloride (DAPI). Scale bar: 20 µm. *n*  =  10 regions. (G, H) Confocal images (g) and quantification (h) of postsynaptic puncta size (Homer‐1, green) in neuron–glioma co‐cultures. White, MAP2; blue, DAPI. Scale bar: 20 µm. *n*  =  9 regions. (I) Quantification of glioblastoma cell proliferation index, showing a marked decrease in ES‐treated cells co‐cultured with primary mouse hippocampal neurons. *n*  =  10, 3 per group. (J, K) Immunohistochemistry images (j) and quantification (k) of Ki‐67 protein expression in glioblastoma tissues, showing reduced proliferation in the IVT+ES group. Scale bar: 50 µm; *n*  =  10 regions. (L, M) Confocal imaging (l) and quantification (m) of synapsin‐1 puncta within glioblastoma tissue. Synapsin‐1 (red) indicates presynaptic puncta, whereas neurofilament heavy/medium chains (NFH/M, white) identify neurons. Scale bar: 10 µm. Insets highlight magnified synapsin‐1 puncta. Scale bar: 2 µm; *n*  =  21 regions. (N, O) Images (n) and quantification (o) of PSD95 puncta counts in glioblastoma tissue. Red, PSD95 (postsynaptic puncta); white, NFH/M. Scale bar, 10 µm. Inset: magnified PSD95 puncta. Scale bar: 2 µm; *n*  =  10 sections. Statistical significance was analyzed using one‐way ANOVA followed by Dunnett's multiple‐comparisons test. NS, not significant. ^*^ *p* < 0.05, ^**^ *p* < 0.01, ^****^ *p* < 0.0001.

Numerous lines of evidence suggest that synaptic connections arise between glioma cells and neurons within the TME [5, 51]. Given the established role of synaptogenic factors in neuron‐to‐glioma synapse formation [5], we hypothesize that the eye‐brain electrical stimulation circuit suppresses the expression of glioma synaptogenic factors, thereby disrupting the establishment of neuron‐to‐glioma synapses and contributing to the observed antitumor effects. Considering the neuronal tropism exhibited by glioma cells, their integration into neural networks requires tumor cells to invade the brain parenchyma and colocalize with neuronal elements [52]. To further examine this phenomenon, we assessed the influence of neuron‐conditioned medium (NCM) on glioma cell invasion under electrically stimulated and non‐stimulated conditions (Figure 3A). Scanning electron microscopy (SEM) analysis was performed on glioma cells exposed to either ES or non‐stimulation, under conditions with or without NCM. Interestingly, the non‐stimulation group with NCM showed pronounced cytoplasmic extensions linking glioma cells. However, in the ES group with NCM, both invasive capacity and microtube length were significantly reduced in glioma cell cultures (Figure 3B–D), highlighting the inhibitory effect of ES on glioma‐neuron integration.

To validate this hypothesis and explore the underlying mechanisms, we performed in vitro co‐culture assays to assess the effects of ES on glioma‐neuron synaptic connectivity. Glioma cells were co‐cultured with primary mouse hippocampal neurons, after which the number of colocalized pre‐ and postsynaptic puncta labeled by synapsin‐1 and the size of postsynaptic structures marked by Homer‐1 were quantified. ES treatment markedly reduced the number of colocalization events and decreased the size of Homer‐1^+^ postsynaptic puncta within glioma cells and neuronal processes compared with untreated co‐cultures (Figure 3E–H and Figure S22). To further support the functional relevance of this reduced synaptic connectivity, we performed live‐cell Ca^2^^+^ imaging in neuron–GL261 co‐cultures. Neuronal co‐culture markedly enhanced GL261 Ca^2^^+^ responses, whereas ES significantly attenuated this neuron‐driven response. CNQX/APV blockade produced a similar reduction, supporting the involvement of glutamatergic neuron‐to‐glioma communication (Figure S23). Together, these structural and functional data suggest that ES reduces glioma‐neuron synaptic connectivity and weakens neuron‐driven glioma Ca^2^^+^ activity.

To assess whether ES affects the proliferation of glioma cells differently in response to neuron‐derived factors relative to glioma cells under standard culture conditions, glioma cells were cultured either alone or together with mouse hippocampal neurons, and co‐culture with 5‐ethynyl‐2′‐deoxyuridine (EdU) overnight. Importantly, ES treatment of GL261 cells in monoculture did not significantly alter proliferation or viability under the tested conditions (Figures S24 and S25), and the results showed that glioma cell proliferation increased by 1.2‐fold when co‐cultured with neurons, indicating a strong dependency on neuronal factors. In contrast, the proliferation index of glioma cells subjected to ES, defined as the fraction of DAPI‐labeled cells co‐expressing EdU, significantly decreased in the presence of mouse hippocampal neurons (Figure 3I and Figure S26). This suggests that ES weakens interaction between neurons and glioma cells, thereby reducing glioma cell proliferation. Consistent with this observation, we found that mice treated with IVT+ES presented reduced Ki‐67 proliferative marker staining within tumor regions, indicating diminished glioma proliferation (Figure 3J,K).

Finally, we analyzed tumor regions of GL261 glioblastoma‐bearing mice subjected to IVT+ES treatment and untreated controls using immunohistochemistry and confocal microscopy [53]. Quantitative analysis demonstrated a reduction in presynaptic puncta labeled by synapsin‐1, together with decreased postsynaptic puncta density, smaller PSD95^+^ neurofilament^+^ neuronal clusters, and reduced synapsin–PSD95 colocalization (Figure 3L–O) within tumor regions relative to untreated controls. These findings indicate reduced synapse stability and formation in glioblastoma, demonstrating the involvement of ES in glioma‐associated neural circuit remodeling and its contribution to tumor progression inhibition.

### 2.4 Disruption of Pathological Neuron Connectivity and Remodeling of Neural Networks

**FIGURE 4:**
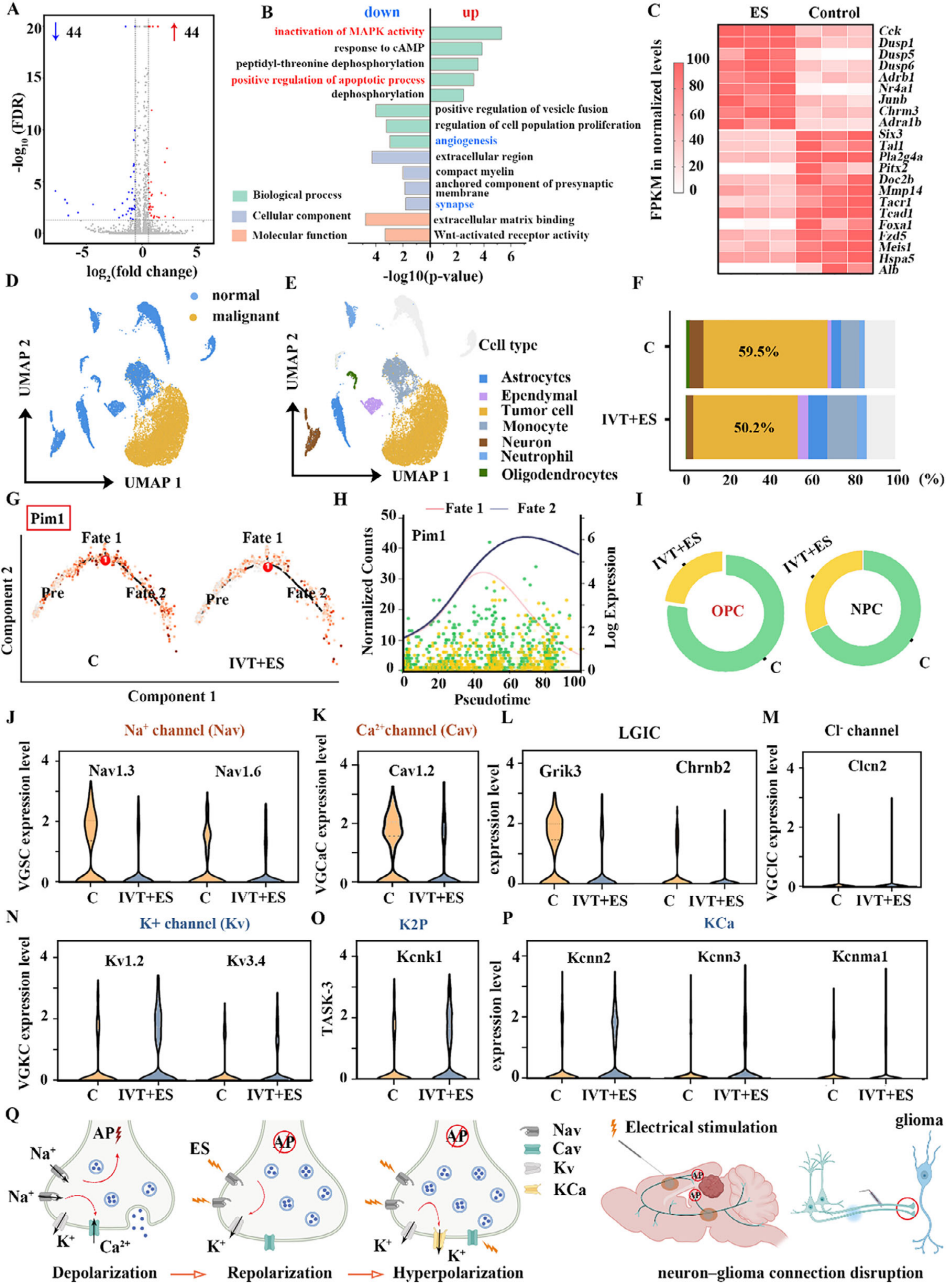
ES inhibits neuron‐to‐glioma synapse formation, disrupts neuronal excitability, and suppresses glioblastoma progression. (A) Volcano plot of the corresponding gene expression from the IVT+ES‐treated mice compared with that of control mice (*p* < 0.05 and |log_2_(fold change)| > 1; *FDR*, *p*‐value adjusted for multiple hypothesis testing). (B) Gene Ontology (GO) enrichment analysis of differentially expressed genes in glioma from IVT+ES‐treated mice, including both upregulated and downregulated genes. (C) Heatmap illustrating genes involved in immune‐related pathways and synapse formation. FPKM, fragments per kilobase of transcript per million mapped reads. (D) UMAP visualizations for normal (blue) and malignant (orange) cells determined by inferCNV analysis. (E) UMAP visualizations show cell populations. Dots representative of individual cells are colored by population. (F) Frequency distribution of cell populations from (e). (G) Pseudotime trajectory analysis of *Pim1* in tumor cell populations following IVT+ES and control treatments. (H) Monocle‐generated plots illustrating the pseudotime‐ordered expression of selected marker gene *Pim1*. (I) Donut charts showing the proportions of oligodendrocyte progenitor cells (OPC) and neural progenitor cells (NPC) in control and IVT+ES groups, respectively. (J–P) Violin plot of (j) voltage‐gated Na^+^ channels gene expression (Nav); (k) voltage‐gated Ca^2+^ channels gene expression (Cav); (l) Ligand‐gated ion channel gene expression (LGIC); (m) voltage‐gated Cl^−^ channels gene expression; (n) voltage‐gated K^+^ channels gene expression (Kv); (o) Two‐pore domain K^+^ channels (K2P) gene expression and (p) Calcium‐activated K^+^ channels (KCa) gene expression. (Q) Schematic illustration showing the mechanism by which ES interferes with neuronal excitability and neuron‐to‐glioma communication.

Based on the above findings that ES suppresses the expression of synaptogenic factors, we further investigated how ES influences tumor‐associated synaptic gene expression using transcriptome sequencing of post‐treatment tumor tissues. This approach identified 88 differentially expressed genes, including 44 upregulated and 44 downregulated transcripts in tumors from IVT+ES‐trained mice relative to control mice, compared with those from the control mice (Figure 4A). GO enrichment analysis (Figure 4B,C) further indicated that genes upregulated in IVT+ES‐trained mice were enriched in signal‐transduction pathways, including inactivation of the MAPK signaling pathway (*Dusp1, Dusp6, Dusp5*), response to cAMP pathway (*Dusp1*), protein dephosphorylation (*Dusp1, Dusp5)*, apoptotic pathways (*Junb*), and other related processes. Downregulated genes were enriched in the angiogenesis pathway (*Mmp14, Tcf7*) and synapse formation suppression. Collectively, these results highlight that IVT+ES disrupts tumor‐associated synaptic pathways and angiogenesis, providing a novel strategy for glioma suppression by targeting neuron‐glioma interactions.

To investigate the molecular and cellular mechanisms underlying neuron‐associated glioma, we performed single‐cell RNA sequencing (scRNA‐seq) on tumor samples. A total of 20 105 high‐quality cells were analyzed, and copy‐number variations (CNVs) were inferred using the inferCNV algorithm to identify malignant cells [54] (Figure 4D). Unsupervised clustering revealed major cellular classes (Figure 4E), with a significant increase in myeloid lineage cells, particularly monocytes, in the IVT+ES group (14.3%) compared to the non‐treated group (8.7%). This increase was associated with upregulated immune‐ associated functions, including antigen presentation and cytokine production, further supporting the hypothesis that IVT+ES enhances immune activation within the TME. Importantly, the fraction of malignant tumor cells in the IVT+ES group (50.2%) was markedly reduced relative to the control group (59.5%) (Figure 4F). In order to gain deeper insights into intratumoral heterogeneity, tumor cells were divided into distinct subclusters through unsupervised clustering analysis (Figure S27). The IVT+ES group manifested a significant reduction in clusters 0, 1, and 2—characterized by enhanced proliferation and immunosuppressive functions‐ and enrichment in clusters 3, 4, and 5, which displayed upregulated immune‐related pathways, including immune response and antigen processing (Figure S28). These findings suggest that IVT+ES treatment reprograms the intratumoral cell population from a proliferative and immunosuppressive state to an immune‐activated state, potentially contributing to improved therapeutic outcomes. Furthermore, Pim1, a serine/threonine kinase critical for tumor progression, was identified as a key regulator in these changes. Pseudotime analysis illustrated a gradual upregulation of Pim1 expression over time in the control group, which was markedly reduced in the IVT+ES group, suggesting that IVT+ES inhibits cellular differentiation and tumor progression (Figure 4G,H).

Previous studies have shown that oligodendrocyte progenitor cells (OPCs) modulate synaptic connectivity and exploit neurodevelopmental features, playing a central role in electrochemical networks and glioma progression [55]. In our analysis, tumor samples from the IVT+ES group displayed significantly lower proportions of OPCs (IVT+ES: 22.7% vs. Control: 77.3%) and neural progenitor cells (NPCs) (IVT+ES: 31.9% vs. Control: 68.1%), underscoring the impact of IVT+ES treatment on reducing tumor‐neuron interactions (Figure 4I and Figure S29). To further evaluate the effects of ES on neuronal excitability, we analyzed the expression levels of sodium (Na^+^), calcium (Ca^2^^+^), potassium (K^+^), and ligand‐gated ion channels (LGIC) in the control and IVT+ES groups [54]. The IVT+ES group exhibited significant downregulation of Na^+^ channels (Nav1.3 and Nav1.6) and Ca^2^^+^ channels (Cav1.2), which likely suppress cellular depolarization and reduce excitability in both neurons and tumor cells (Figure 4J–L). In addition, LGIC, including Grik3 and Chrnb2, were downregulated, indicating that IVT+ES treatment may inhibit excitatory synaptic transmission (Figure 4L). Conversely, the expression of Cl^−^ channels (Clcn2), K^+^ channels (Kv1.2, Kv3.4), K2P channels (Kcnk1), and KCa channels (Kcnn2, Kcnn3, Kcnma1) was upregulated (Figure 4M–P), which promotes cellular repolarization and reduces excitability. Functionally, Fluo‐4 AM Ca^2^^+^ imaging further showed that ES attenuated basal and GL261‐enhanced neuronal Ca^2^^+^ responses, similar to the effect of the voltage‐gated Na^+^ channel blocker TTX, supporting ES‐associated reduction of neuronal activity (Figure S30). Collectively, these findings suggest that IVT+ES treatment modulates neuronal excitability‐associated pathways and neuronal Ca^2^^+^ activity within the tumor microenvironment, contributing to its antitumor effects by disrupting pathological neuronal activity.

Mechanistically, in Figure 4Q, neuronal depolarization is facilitated by sodium (Na^+^) and calcium (Ca^2^^+^) influx through voltage‐gated sodium (Nav) and calcium (Cav) channels under normal conditions, resulting in action potential (AP) generation and synaptic transmission. ES is proposed to attenuate AP‐associated neuronal depolarization by enhancing potassium (K^+^) efflux via voltage‐gated potassium channels (Kv) together with calcium‐activated potassium channels (KCa), leading to neuronal repolarization and subsequent hyperpolarization. In summary, ES applied to the eye‐brain neuroimmune axis disrupts neuron‐glioma synaptic integration, thereby inhibiting glioma progression by reducing neuronal excitability and neuron‐to‐glioma communication.

### 2.5 Eye‐Brain Electrical Stimulation Circuit Effectively Inhibits Glioma Growth

**FIGURE 5:**
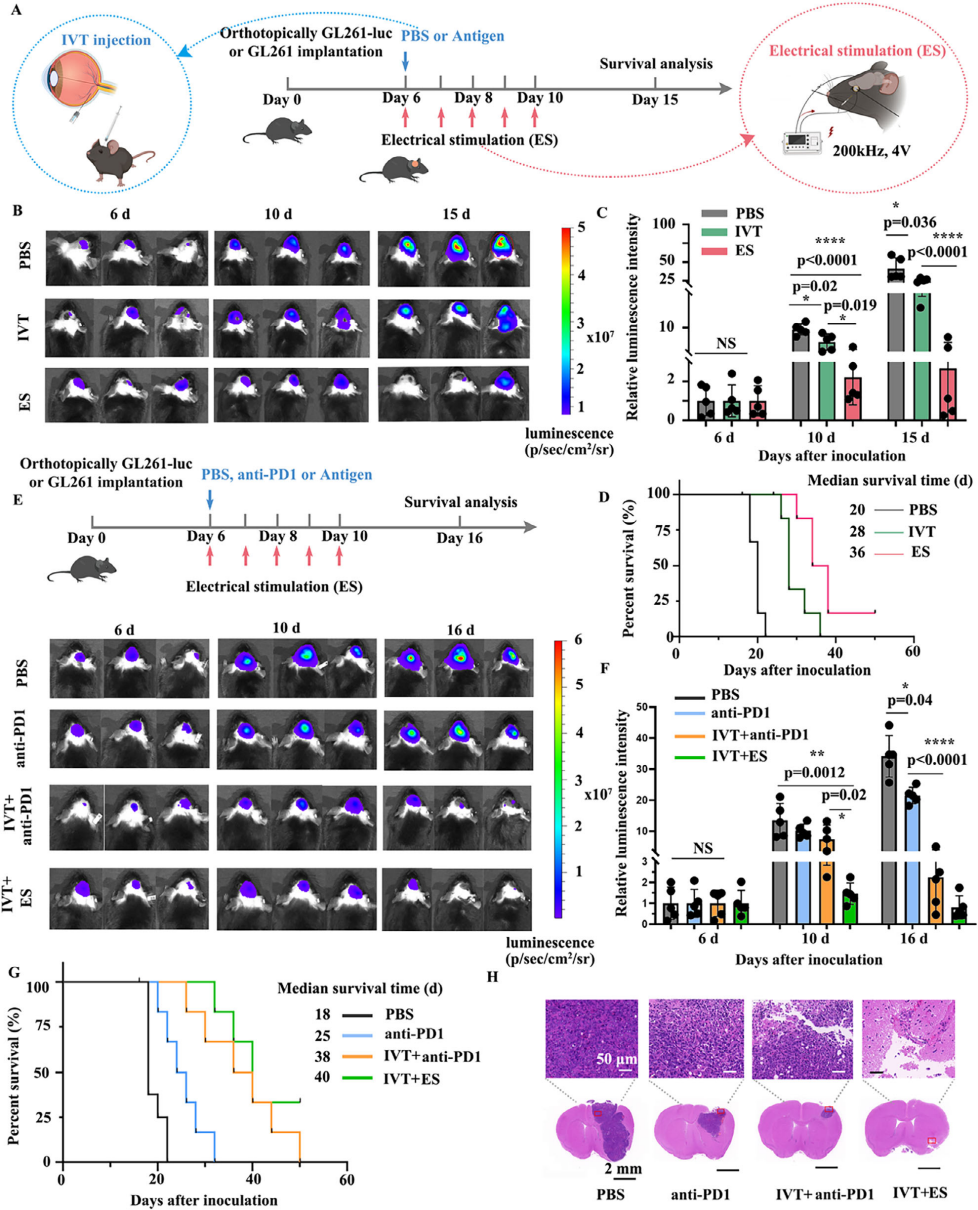
ES of the eye‐brain neuroimmune axis suppressed tumor growth and extended survival in mice bearing intracranial GL261 GBM. (A) An overview of the experimental schedule is described below. IVT: Mice bearing intracranial GL261‐luc GBM were immunized using irradiated GL261–Luc cells (serving as antigen) through IVT administration (day 6). ES: Mice bearing intracranial tumors were treated via the non‐invasive brain‐eye electrical stimulation circuit for 5 consecutive days (from day 6 to day 10), with stimulation at 200 kHz for 5 min per session. (B) In vivo bioluminescence imaging of intracranial tumor‐bearing mice after different treatments (D6, D10, and D15). (C) Semi‐quantitative analysis of tumor burden derived from the bioluminescence signals shown in panel b (*n* = 5 mice). (D) Kaplan–Meier survival curves for mice with intracranial GL261 GBM after the indicated treatments (*n* = 6 mice). (E) Schematic representation of the study procedure timeline is outlined below. Anti‐PD1: Mice bearing intracranial tumors were treated with anti‐PD1 via intraperitoneal (*i.p*.) injection (day 6). In vivo bioluminescence imaging of intracranial tumor‐bearing mice after different treatments (D6, D10, and D16). (F) Quantification of tumor burden 6, 10, and 16 days after tumor inoculation (*n* = 5 mice). (G) Kaplan–Meier survival analysis of intracranial GL261 GBM‐bearing mice under different treatment conditions (*n* = 6 mice). (H) H&E staining of intracranial GL261 GBM sections collected on day 16 after tumor inoculation. Statistical significance was analyzed using one‐way ANOVA followed by Tukey's multiple‐comparisons test. Adjusted *p*‐values for the indicated comparisons are shown in the figure. NS, not significant. ^*^p < 0.05, ^**^p < 0.01, ^***^p < 0.001, ^****^p < 0.0001.

The intricate connection linking the eye and the brain plays a crucial role in both neural regulation and immune modulation, which is essential for understanding and treating brain disorders [56]. In this study, we employed a non‐invasive brain‐eye electrical stimulation (ES) circuit to establish a functional link between the brain and eye, aiming to harness its dual potential to activate immune responses and remodel aberrant neural circuits. Based on an in vivo frequency‐screening experiment, 200 kHz was selected as the working condition for subsequent studies (Table S1). We evaluated its effects on tumor growth inhibition via both enhanced immune activation and disruption of pathological neural connectivity. Additionally, intravitreal (IVT) injection of irradiated GL261‐Luc cells was employed to further stimulate antigen‐specific immune responses against intracranial glioma, thereby synergizing with ES to effectively target the tumor microenvironment (Figure 5A). In vitro and in vivo safety evaluations demonstrated that high‐frequency ES does not cause detectable ocular damage. In addition, open‐field, rotarod, and novel object recognition tests showed no obvious deficits in locomotion, motor coordination, or recognition memory (Figures S31–S35). Together, these findings establish a solid safety profile for the non‐invasive ES approach, paving the way for its therapeutic application in glioblastoma. As shown in Figure 5B, high‐frequency (i.e., 200 kHz) ES impressively suppressed rapid tumor growth [54, 57] relative to the PBS group, as indicated by reduced tumoral luciferase intensity and H&E staining (Figure S36a). Notably, while IVT alone demonstrated a partial inhibitory effect (Figure 5B,C), the combination of ES and IVT illustrated a more pronounced antitumor response, suggesting that both immune activation and neural circuit remodeling contribute to tumor suppression. ES‐treated mice exhibited significantly prolonged survival (median of 36 days) compared to IVT‐treated (28 days) or PBS‐treated mice (20 days) (Figure 5D). Moreover, 16.7% of the ES‐treated mice were tumor‐free with no recurrence observed until day 50, and no significant loss of body weight was observed, showcasing the efficacy and tolerability of the combined immunomodulatory and neural remodeling effects (Figure S36b).

Encouraged by the robust antitumor effect of ES, we further investigated the therapeutic efficacy of ES combined with IVT using a GL261 orthotopic model (derived from C57BL/6 mice). For comprehensive comparison, anti‐PD1 treatment alone and in combination with IVT were used as controls to delineate the contributions of immune modulation vs. tumor‐neural network disruption (Figure 5E). Tumor progression was markedly inhibited following IVT+ES treatment, as demonstrated by a 98.3% reduction in luciferase intensity compared to the control group, whereas the anti‐PD1 treatment alone revealed minimal inhibition [58], with results similar to those of the anti‐PD1+IVT group and the control on day 10 (Figure 5F). The IVT+ES treatment significantly extended survival (median survival: 40 days), with 33.3% of mice surviving up to day 50, compared to a median survival of 38 days in the anti‐PD1+IVT group and only 25 days in the anti‐PD1 group (Figure 5G). These findings were further supported by brain H&E staining (Figure 5H). Comparable results were witnessed in the orthotopic glioma model established in BALB/c mice, where the IVT+ES treatment group exhibited the most effective inhibition of glioma growth (Figures S37 and S38).

We further established the orthotopic glioma model in nude mice, which are known for their lack of a thymus and inability to produce T cells [59, 60]. In this T cell‐deficient model, the IVT group showed minimal tumor inhibition compared to controls, whereas both the ES and IVT+ES treatment groups exhibited a marked tumor‐suppressive effect (Figures S39 and S40). To further distinguish the neural contribution of ES from adaptive immune activation, we quantified ES‐alone efficacy in nude mice. ES reduced intracranial tumor burden, prolonged median survival from 24 to 30 days, and decreased Synapsin‐1/NFH/M and PSD95/NFH/M puncta ratios in tumor regions, indicating attenuated glioma‐associated synaptic connectivity (Figure S41). These findings suggest that ES‐mediated neural modulation partially suppresses glioma progression independently of a fully competent adaptive immune system. Together with the stronger efficacy observed in immunocompetent mice receiving IVT+ES, these results support a cooperative immune–neural mechanism underlying the overall antitumor effect.

## 3 Conclusions

The eye, as a structural extension of the brain, shares significant similarities with the central nervous system (CNS) in both neural and immune functions, emphasizing the crucial role of the eye‐brain neuroimmune axis. Despite this intimate relationship, previous studies have predominantly focused on these systems independently, without fully exploring their integrated interactions. Recent research has uncovered a common lymphatic pathway linking the posterior eye to the brain, enabling a coordinated immune response and reinforcing the concept of eye‐brain immunity. Nevertheless, the integrated immunological and neurological connection between the eye and CNS remains unexplored. To address this gap, we provide functional evidence supporting an eye–brain neuroimmune axis by systematically analyzing both immune and neuronal responses to eye‐brain electrical stimulation (ES). Our results indicate that ES‐enhanced intravitreal immunization (IVT) facilitates both therapeutic immune responses and neuronal signal modulation. This discovery of the eye‐brain connection offers a promising avenue for therapeutic intervention in neuroimmune‐related diseases.

As demonstrated in our proof of concept, we applied this for glioblastoma treatment, where the vitreous body serves as a non‐invasive, accessible route for antigen delivery. To validate the immunological basis of the eye‐brain neuroimmune axis, we conducted single‐cell RNA sequencing and flow cytometry to assess changes in immune composition in the TME. Our findings revealed that ES accelerates the rapid drainage of intravitreally delivered antigens to dCLNs, effectively bypassing the BBB and triggering robust CNS‐specific immune responses in IVT+ES‐treated mice. Mechanistically, in vitro glioma‐neuron co‐culture experiments conveyed that ES markedly reduced colocalized pre‐ and postsynaptic puncta and decreased the size of postsynaptic clusters. These disruptions weakened glioma‐neuron interactions, resulting in a significant reduction in tumor progression. Consistent with these observations, in vivo IVT+ES treatment resulted in the lowest luciferase intensity (a 98.3% reduction relative to the control group) and tumor burden (33.3% of the mice survived up to day 50; thus far, few studies have documented survival exceeding this threshold in comparable experimental settings [5]).

Despite the promise of the eye‐brain neuroimmune axis approach, several challenges remain. A limitation of the present study is that the ES parameters were selected based on the frequency‐screening experiment, and further optimization is needed. Another limitation is that the mechanistic and therapeutic evaluations were primarily performed in the GL261 model, which may not fully recapitulate the biological heterogeneity and immunosuppressive features of human glioblastoma. Immune responses may vary depending on the type of disease or pathogen, complicating the development of universal immune modulation strategies. For instance, glioblastoma and neurodegenerative diseases may trigger distinct immune profiles, necessitating tailored approaches for optimal immune activation. Moreover, the interaction between immune responses and neuronal activity is not fully understood, particularly in chronic diseases where neuroinflammation is prevalent. The precise impact of immune activation on neuronal signaling and the mechanisms through which immune responses modulate neural circuits need further investigation. Future studies should focus on elucidating the molecular mechanisms underlying this neuroimmune interaction and exploring the clinical potential.

In conclusion, our findings emphasize the critical role of the eye‐brain neuroimmune axis in regulating immune responses and neuronal activity, providing a promising strategy for treating eye‐brain diseases. This approach could extend beyond glioma therapy, offering valuable insights into therapeutic approaches for other neuroimmune‐related brain disorders, including Alzheimer's disease and Parkinson's disease, as well as retinal conditions such as autoimmune retinopathy and choroidal neovascularization. By facilitating both immune activation and neuronal signal modulation, this theoretical framework may represent a paradigm shift in therapeutic strategies. Looking forward, we believe that these insights into the underlying pathophysiology of eye‐brain diseases will guide the development of precise diagnostics and innovative treatments for both ocular and CNS diseases.

## 4 Materials and Methods

The antibodies employed in this study are listed in Table S1.

### 4.1 Mice and Housing Conditions

All animal experiments were conducted in accordance with protocols approved by the Laboratory Animal Center of Soochow University (approval number: SYXK(Su) 2021‐0073) and complied with institutional guidelines for animal care and use. Male and female BALB/c mice (3–5 weeks old), C57BL/6 mice (3–5 weeks old), and nude mice (3–5 weeks old) were obtained from Changzhou Cavens Experimental Animals (China). For brain tumor xenograft experiments, the Institutional Animal Care and Use Committee (IACUC) of Soochow University does not define a strict upper limit for tumor volume but requires close monitoring of animal health and morbidity. Mice were humanely euthanized if they developed neurological symptoms or experienced a body weight loss of 15% or greater. Kaplan–Meier survival curves were analyzed using the log‐rank test to determine statistical significance.

### 4.2 Orthotopic Xenografting

Male and female mice (postnatal day 28–30) were used for orthotopic glioma implantation. Animals were anesthetized with 1–4% isoflurane and secured in a stereotactic frame. After exposing the skull via a midline incision under sterile conditions, GL261–Luc cells (approximately 6 × 10^5^ cells in 3 µL sterile PBS) were stereotactically delivered into the premotor cortex through a 31‐gauge burr hole. Cell suspensions were injected using a Hamilton syringe connected to a digital pump at a rate of 0.4 µL min^−^^1^. The stereotactic coordinates were 0.5 mm lateral to the midline, 1.0 mm posterior to the bregma, and 1.75 mm below the cranial surface. After injection, the needle remained in place for 2 min to minimize reflux and was then slowly withdrawn at 0.875 mm min^−^^1^.

### 4.3 IVT Immunization

Mice were anesthetized by intraperitoneal administration of ketamine (50 mg kg^−^^1^) and xylazine (5 mg kg^−^^1^). Prior to intravitreal injection, the pupils were dilated using tropicamide ophthalmic solution. A 30‐gauge needle was used to create an entry point about 1 mm behind the corneoscleral junction. A blunt‐tip Hamilton syringe loaded with 1 µL dye or irradiated tumor cells was gently advanced into the vitreous cavity to a depth of 1–2 mm, and the solution was delivered at a rate of 1 µL min^−^^1^. To prevent cataract development, petrolatum ophthalmic ointment was applied to the eye immediately after injection. Mice were then kept on heating pads and observed until complete recovery from anesthesia.

### 4.4 Eye‐Brain Electrical Stimulation Circuit

The eye–brain electrical stimulation (ES) system used in this study was structurally simple. An active electrode was positioned on the corneal surface, while a reference electrode was inserted beneath the scalp overlying the brain. Electrical stimulation was generated using a pulse generator connected to the active electrode, enabling controlled current delivery. The stimulation parameters were set as follows: current amplitude (A): ±4 V; stimulation frequency (f): 50 kHz, 200 and 300 kHz.

### 4.5 In vivo Tumor Treatment and IVIS Imaging

Tumor‐bearing mice were randomly allocated into six experimental groups: (I) blank control, (II) IVT, (III) ES, (IV) anti‐PD1 (i.p. injection, 5 mg/kg), (V) IVT+ES, and (VI) IVT+anti‐PD1. Tumor progression was monitored every two days using bioluminescence imaging with the IVIS Spectrum In Vivo Imaging System (PerkinElmer). For imaging, mice were anesthetized with isoflurane and injected intraperitoneally with D‐luciferin potassium salt (PerkinElmer; 200 µL, 30 mg mL^−^^1^). Bioluminescence signals were recorded 10 min after substrate administration and quantified to evaluate tumor burden.

### 4.6 Imaging and Quantification of Tracer Transport

To quantify fluorescence signals in the eye and lymph nodes (LNs), 70 kDa FITC‐dextran was administered via intravitreal injection into the vitreous body. Following injection, mice in the stimulation group received 5 min of electrical stimulation. To evaluate tracer transport, the eye, superficial cervical lymph nodes (sCLNs), and deep cervical lymph nodes (dCLNs) were collected after either IVT alone or IVT+ES treatment. Collected tissues were subsequently imaged using the IVIS Spectrum In Vivo Imaging System (PerkinElmer).

### 4.7 Perfusion, Immunohistochemistry and Immunofluorescence Analysis

Mice were anesthetized via intraperitoneal administration of avertin (tribromoethanol) and perfused transcardially with 20 mL PBS. Brains were collected and fixed overnight in 4% paraformaldehyde (PFA) at 4°C, followed by cryoprotection in 30% sucrose solution. Samples were embedded in Tissue‐Tek O.C.T. compound (Sakura) and sectioned coronally at 40 µm using a sliding microtome (Microm HM450; Thermo Scientific).

For immunofluorescence staining, 5 µm paraffin‐embedded sections were subjected to antigen retrieval, then blocked and incubated with primary antibodies overnight at 4°C. Primary antibodies included (all used at 1:500, Invitrogen): rabbit anti‐synapsin‐1 (1:500, UpingBio); rabbit anti‐PSD95 (1:100, UpingBio); rabbit anti‐neurofilament (M+H) (1:500, UpingBio) and rabbit anti‐Ki‐67 (1:100, Abcam). Species‐specific secondary antibodies were applied: Alexa Fluor 488 goat anti‐rabbit IgG; Alexa Fluor 594 goat anti‐rabbit IgG, and Alexa Fluor 647 goat anti‐rabbit IgG. After DAPI nuclear staining (1:100, Beyotime), slides were mounted using Fluoromount‐G mounting medium (Southern Biotech) for imaging.

Synapsin‐1 and PSD95 puncta were quantified using the spot detection module in Imaris software, with automatic intensity thresholding and a spot diameter of 1.0 µm. The ratio of pre‐ to postsynaptic puncta was determined by calculating the number of synapsin‐1– or PSD95–positive puncta located on neurofilament‐positive neurons relative to the total number of DAPI‐labeled cells within imaging fields of 135 µm × 135 µm.

For immunohistochemical analysis, tissue sections were subjected to DAB‐based chromogenic detection using horseradish peroxidase (Vector Laboratories), followed by incubation with ImmPress anti‐rabbit IgG (Novus Biologicals) and counterstaining with Harris hematoxylin.

### 4.8 Single‐Cell Sequencing

#### 4.8.1 Single‐Cell Suspension Generation

Fresh tumor tissues and dCLNs were collected and transported on ice in PBS. Tumor samples were minced (10 scalpels) (Integra LifeSciences) and digested in 1 mg mL^−^^1^ papain (Miltenyi Biotec) at 37°C for 45 min. Following digestion, red blood cells were removed using RBC lysis buffer (Miltenyi Biotec) for 10 min at room temperature. The remaining cell pellets were resuspended in PBS containing 0.04% BSA and passed through a 35 µm cell strainer. Cell viability was evaluated using AO/PI staining and quantified with a Countstar Fluorescence Cell Analyzer.

#### 4.8.2 Single‐Cell Sequencing and Analysis

Single‐cell RNA sequencing was performed using the NovelCyto Single‐Cell Analysis System (NovelBio, China). Single‐cell suspensions were loaded into >100 000 microwells by limiting dilution, followed by the addition of oligonucleotide‐barcoded beads to capture individual cells. After cell lysis within microwells, released mRNA molecules hybridized to bead‐bound capture oligos. The beads were subsequently pooled into a single tube for reverse transcription and ExoI digestion. During cDNA synthesis, each transcript was labeled with a unique molecular identifier (UMI) and a cell‐specific barcode, enabling transcript quantification at the single‐cell level.

Whole‐transcriptome amplification libraries were generated using the NovelCyto single‐cell WTA workflow, which includes random priming, extension, PCR amplification, and index PCR. Library quality was assessed using an Agilent Bioanalyzer 4200 High Sensitivity DNA chip and Qubit High Sensitivity DNA assay. Sequencing was performed on the DNBSEQ‐T7 platform (MGI) with 150 bp paired‐end reads. Single‐cell RNA‐seq data were processed using the NovelBrain Cloud Analysis Platform. Adapter trimming and quality filtering were performed using fastp. Cell barcode whitelists were identified using UMI‐tools. Clean reads were mapped to the mouse genome (mm10, Ensembl v100) using STAR, with customized parameters derived from the UMI‐tools pipeline to generate UMI count matrices [61].

### 4.9 Glioma–Hippocampal Neuron Co‐Culture Model

GL261 cells were first seeded on poly‐D‐lysine/laminin‐coated coverslips (Neuvitro) at 1 × 10^4^ cells per well in 24‐well plates. After 24 h, 4 × 10^4^ embryonic mouse hippocampal neurons were added onto the GL261 layer. The co‐cultures were maintained in serum‐free Neurobasal medium containing B27, gentamicin, and GlutaMAX (Gibco). After two weeks, cultures were fixed with 4% paraformaldehyde (PFA) for 30 min at 4°C. Samples were subsequently blocked for 1 h at room temperature in PBS containing 5% normal donkey and goat serum together with 0.25% Triton X‐100. Primary antibodies prepared in blocking buffer were applied overnight at 4°C, including rabbit anti‐Homer‐1 (1:250, UpingBiotech), rabbit anti‐synapsin‐1 (1:200), and pig anti‐MAP2 (1:500, Abcam). After primary antibody incubation, coverslips were rinsed three times with PBS and incubated with secondary antibodies (Alexa Fluor 488 goat anti‐pig IgG, Alexa Fluor 568 goat anti‐rabbit IgG, and Alexa Fluor 647 goat anti‐rabbit IgG; 1:500, Invitrogen) in antibody diluent for 1 h at room temperature. Finally, coverslips were washed with PBS and mounted using antifade mounting medium containing DAPI for fluorescence imaging.

### 4.10 Confocal Imaging and Quantitative Analysis of Synapsin‐1 and Homer‐1 Colocalization

Confocal fluorescence images were acquired at 1024 × 1024 resolution using a 10 × objective on a Nikon C2 confocal microscope. Imaging parameters for the Homer‐1 Alexa488 and synapsin‐1 Alexa647 channels were maintained identical across all experimental samples. The acquired image stacks were imported into Imaris software (v9.2.1, Bitplane) for analysis. Fluorescence intensity thresholds for each channel were manually defined, and colocalization between synapsin‐1 puncta and Homer‐1 signals was identified by generating a colocalization channel using the Imaris colocalization module. Colocalization events were subsequently quantified using the Imaris spot detection algorithm together with the colocalization channel. MAP2‐labeled dendrites were visualized in the TRITC channel and reconstructed using the Filament module of Imaris. Synaptic puncta showing synapsin‐1 and Homer‐1 colocalization were counted and normalized to dendrite length (puncta per 10 µm dendrite). Homer‐1–positive puncta were generated using the Imaris Surface module from maximal intensity projection images. Surfaces were generated with a surface detail parameter of 0.1 µm and thresholded by absolute fluorescence intensity, including voxels with values >1.0.

The area of individual Homer‐1–positive puncta was measured, and the mean puncta size was calculated for quantitative comparison.

### 4.11 Scanning Electron Microscopy (SEM) Analysis

SEM was used to examine tumor microtube structures at high resolution. GL261 cells were plated on poly‐D‐lysine/laminin‐coated coverslips (Neuvitro) at 1 × 10^4^ cells per well in 24‐well plates. For glioma‐only and glioma + ES groups, mouse neuron‐conditioned medium was introduced into the wells with or without electrical stimulation, respectively, to evaluate the influence of neuron‐derived factors on tumor microtube formation in glioma cells. After one week of culture, cells were fixed with 4% paraformaldehyde (PFA) for 30 min at 4°C, followed by rinsing with PBS and ultrapure water. Specimens were dehydrated through a graded ethanol series, after which coverslips were attached to SEM stubs (TED Pella) using conductive carbon adhesive tabs (12 mm OD PELCO Tabs, TED Pella). The mounted specimens were then sputter‐coated with a ∼2 nm gold–palladium layer prior to imaging. Tumor microtubes were visualized using a field‐emission scanning electron microscope (Sigma 500, Carl Zeiss), and images were acquired at an accelerating voltage of 1.0 kV.

## Funding

We appreciate the financial support from the National Natural Science Foundation of China (T2321005, 22393932, and 22504095), the Science and Technology Development Fund, Macau SAR (grant number: 0002/2022/AKP, 0115/2023/RIA2), the National Key R&D Program of China (2023YFB3208202), the Natural Science Foundation of Jiangsu Province of China (BK20240766), the 111 Project and the Collaborative Innovation Center of Suzhou Nano Science and Technology (NANO‐CIC). We appreciate the financial support from the Excellent Postdoctoral Program of Jiangsu Province, the Postdoctoral Fellowship Program of CPSF (GZB20230501), and the Postdoctoral Science Foundation of China (2023M742532, 2024T170625).

## Conflicts of Interest

The authors declare no conflicts of interest.

## Supporting information




**Supporting File**: advs76265‐sup‐0001‐SuppMat.docx.

## Data Availability

The data that support the findings of this study are available from the corresponding author upon reasonable request.

## References

[1] J. Bolaños‐Cardet , S. Pugliese , J. Bruna , D. Ruiz‐Molina , S. Suárez‐García , and V. J. Yuste , “A Mussel‐Inspired Bioadhesive Patch to Selectively Kill Glioblastoma Cells,” Advanced Science 13 (2026): 10658, 10.1002/advs.202510658. PMC13088311 41591765

[2] X. An , Y. Guo , Y. Bian , et al., “A Cascade Recognition of Activatable Probe for Fluorescence Navigation Glioblastoma Surgery: Overcoming Blood‐Brain Barrier and Off‐Target Limitations,” Advanced Science 13 (2026): 21404, 10.1002/advs.202521404. PMC13088282 41665406

[3] L. Starck , T. Butelmann , S. Hogan , et al., “Stochastic Nanoscale Biophysical Cues as a Basis for the Induction of Glioblastoma‐Like Transcriptional Programs in Astrocytes,” Advanced Science 13 (2026): 09362, 10.1002/advs.202509362. PMC13067847 41632084

[4] S. Singh , D. Dey , D. Barik , et al., “Glioblastoma at the Crossroads: Current Understanding and Future Therapeutic Horizons,” Signal Transduction and Targeted Therapy 10 (2025): 213, 10.1038/s41392-025-02299-4. 40628732 PMC12238593

[5] H. S. Venkatesh , W. Morishita , A. C. Geraghty , et al., “Electrical and Synaptic Integration of Glioma Into Neural Circuits,” Nature 573 (2019): 539–545, 10.1038/s41586-019-1563-y. 31534222 PMC7038898

[6] C. Dmello , J. Zhao , L. Chen , et al., “Checkpoint Kinase 1/2 Inhibition Potentiates Anti‐Tumoral Immune Response and Sensitizes Gliomas to Immune Checkpoint Blockade,” Nature Communications 14 (2023): 1566, 10.1038/s41467-023-36878-2. PMC10033639 36949040

[7] G. Castellani , T. Croese , J. M. Peralta Ramos , and M. Schwartz , “Transforming the Understanding of Brain Immunity,” Science 380 (2023): 7649, 10.1126/science.abo7649. 37023203

[8] S. Zhou , Y. Huang , Y. Chen , et al., “Reprogramming Systemic And Local Immune Function To Empower Immunotherapy Against Glioblastoma,” Nature Communications 14 (2023): 435, 10.1038/s41467-023-35957-8. PMC9880004 36702831

[9] S. Pei , X. Deng , R. Yang , et al., “Age‐Related Decline in CD^8+^ Tissue Resident Memory T Cells Compromises Antitumor Immunity,” Nature Aging 4 (2024): 1828–1844, 10.1038/s43587-024-00746-5. 39592880

[10] M. W. Kim , W. Gao , C. F. Lichti , et al., “Endogenous Self‐Peptides Guard Immune Privilege of the Central Nervous System,” Nature 637 (2025): 176–183, 10.1038/s41586-024-08279-y. 39476864 PMC11666455

[11] C. Wei , W. Jiang , R. Wang , et al., “Brain Endothelial GSDMD Activation Mediates Inflammatory BBB Breakdown,” Nature 629 (2024): 893–900, 10.1038/s41586-024-07314-2. 38632402

[12] R. D. Leone and J. D. Powell , “Metabolism of Immune Cells in Cancer,” Nature Reviews Cancer 20 (2020): 516–531, 10.1038/s41568-020-0273-y. 32632251 PMC8041116

[13] L. Liu , X. Zhang , Y. Chai , J. Zhang , Q. Deng , and X. Chen , “Skull Bone Marrow and Skull Meninges Channels: Redefining the Landscape of Central Nervous System Immune Surveillance,” Cell Death & Disease 16 (2025): 53, 10.1038/s41419-025-07336-2. 39875352 PMC11775313

[14] A. Louveau , I. Smirnov , T. J. Keyes , et al., “Structural and Functional Features of Central Nervous System Lymphatic Vessels,” Nature 523 (2015): 337–341, 10.1038/nature14432. 26030524 PMC4506234

[15] X. Yin , S. Zhang , J. H. Lee , et al., “Compartmentalized Ocular Lymphatic System Mediates Eye–Brain Immunity,” Nature 628 (2024): 204–211, 10.1038/s41586-024-07130-8. 38418880 PMC10990932

[16] B. Laha , B. K. Stafford , and A. D. Huberman , “Regenerating Optic Pathways From the Eye to the Brain,” Science 356 (2017): 1031–1034, 10.1126/science.aal5060. 28596336 PMC6333302

[17] A. London , I. Benhar , and M. Schwartz , “The Retina As A Window To The Brain—From Eye Research To CNS Disorders,” Nature Reviews Neurology 9 (2013): 44–53, 10.1038/nrneurol.2012.227. 23165340

[18] H. L. Weiner , “Immune Mechanisms and Shared Immune Targets in Neurodegenerative Diseases,” Nature Reviews Neurology 21 (2025): 67–85, 10.1038/s41582-024-01046-7. 39681722

[19] L. Steinman , “Elaborate Interactions Between the Immune and Nervous Systems,” Nature Immunology 5 (2004): 575–581, 10.1038/ni1078. 15164017

[20] T. Du , A. Raghunandan , H. Mestre , et al., “Restoration of Cervical Lymphatic Vessel Function in Aging Rescues Cerebrospinal Fluid Drainage,” Nature Aging 4 (2024): 1418–1431, 10.1038/s43587-024-00691-3. 39147980

[21] C. Chu , D. Artis , and I. M. Chiu , “Neuro‐Immune Interactions in the Tissues,” Immunity 52 (2020): 464–474, 10.1016/j.immuni.2020.02.017. 32187517 PMC10710744

[22] A. Verma , S. K. Agadagba , and L. L. Chan , “Exploring the Synergy of the Eye‐Brain Connection: Neuromodulation Approaches for Neurodegenerative Disorders Through Transcorneal Electrical Stimulation,” Neural Regeneration Research 19 (2024): 2097–2098, 10.4103/1673-5374.392877. 38488536 PMC11034586

[23] V. Chichagova , D. Hallam , J. Collin , et al., “Cellular Regeneration Strategies for Macular Degeneration: Past, Present and Future,” Eye 32 (2018): 946–971, 10.1038/s41433-018-0061-z. 29503449 PMC5944658

[24] B. De Strooper and E. Karran , “The Cellular Phase of Alzheimer's Disease,” Cell 164 (2016): 603–615. 26871627 10.1016/j.cell.2015.12.056

[25] A. Citri and R. C. Malenka , “Synaptic Plasticity: Multiple Forms, Functions, and Mechanisms,” Neuropsychopharmacology 33 (2008): 18–41. 17728696 10.1038/sj.npp.1301559

[26] N. Kiyota , Y. Shinozaki , X. Guo , et al., “Role of HAUS7 as a DOCK3 Binding Partner in Facilitating Axon Regeneration,” Science Advances 11 (2025): adq7105, 10.1126/sciadv.adq7105. PMC12292649 40712007

[27] N. M. Tran , K. Shekhar , I. E. Whitney , et al., “Single‐Cell Profiles of Retinal Ganglion Cells Differing in Resilience to Injury Reveal Neuroprotective Genes,” Neuron 104 (2019): 1039–1055, 10.1016/j.neuron.2019.11.006. 31784286 PMC6923571

[28] B. Zhao , Y. Li , Z. Fan , et al., “Eye‐Brain Connections Revealed by Multimodal Retinal and Brain Imaging Genetics,” Nature Communications 15 (2024): 6064, 10.1038/s41467-024-50309-w. PMC11258354 39025851

[29] J. Hao , W. R. Kwapong , T. Shen , et al., “Early Detection of Dementia Through Retinal Imaging and Trustworthy AI,” NPJ Digital Medicine 7 (2024): 294, 10.1038/s41746-024-01292-5. 39428420 PMC11491446

[30] S. Chiquita , A. C. Rodrigues‐Neves , F. I. Baptista , et al., “The Retina as a Window or Mirror of the Brain Changes Detected in Alzheimer's Disease: Critical Aspects to Unravel,” Molecular Neurobiology 56 (2019): 5416–5435, 10.1007/s12035-018-1461-6. 30612332

[31] S. Sherman , I. Arnold‐Ammer , M. W. Schneider , K. Kawakami , and H. Baier , “Retina‐Derived Signals Control Pace of Neurogenesis in Visual Brain Areas but Not Circuit Assembly,” Nature Communications 14 (2023): 6020, 10.1038/s41467-023-40749-1. PMC10533834 37758715

[32] J. V. Forrester and P. G. McMenamin , “Evolution of the Ocular Immune System,” Eye 39 (2025): 468–477, 10.1038/s41433-024-03512-4. 39653763 PMC11794555

[33] Y. Hu , Y. Lin , L. Cheng , et al., “Hypothesis on the Outflow of Optic Nerve Cerebrospinal Fluid in Spaceflight Associated Neuro Ocular Syndrome,” NPJ Microgravity 10 (2024): 112, 10.1038/s41526-024-00449-6. 39702371 PMC11659609

[34] S. Camelo , J. Kezic , A. Shanley , P. Rigby , and P. G. McMenamin , “Antigen From the Anterior Chamber of the Eye Travels in a Soluble Form to Secondary Lymphoid Organs via Lymphatic and Vascular Routes,” Investigative Opthalmology & Visual Science 47 (2006): 1039–1046, 10.1167/iovs.05-1041. 16505039

[35] A. L. C. Tam , N. Gupta , Z. Zhang , and Y. H. Yücel , “Quantum Dots Trace Lymphatic Drainage From the Mouse Eye,” Nanotechnology 22 (2011): 425101, 10.1088/0957-4484/22/42/425101. 21934199

[36] A. Del Prete , V. Salvi , A. Soriani , et al., “Dendritic Cell Subsets in Cancer Immunity and Tumor Antigen Sensing,” Cellular & Molecular Immunology 20 (2023): 432–447. 36949244 10.1038/s41423-023-00990-6 PMC10203372

[37] T. L. Murphy and K. M. Murphy , “Dendritic Cells in Cancer Immunology,” Cellular & Molecular Immunology 19 (2022): 3–13, 10.1038/s41423-021-00741-5. 34480145 PMC8752832

[38] A. Psarras , A. Alase , A. Antanaviciute , et al., “Functionally Impaired Plasmacytoid Dendritic Cells and Non‐Haematopoietic Sources of Type I Interferon Characterize Human Autoimmunity,” Nature Communications 11 (2020): 6149, 10.1038/s41467-020-19918-z. PMC7708979 33262343

[39] L. López , L. G. Morosi , and F. La Terza , “Dendritic Cell‐Targeted Therapy Expands CD8 T Cell Responses to Bona‐Fide Neoantigens in Lung Tumors,” Nature Communications 15 (2024): 2280. 10.1038/s41467-024-46685-y PMC10937682 38480738

[40] H. Ueno , E. Klechevsky , R. Morita , et al., “Dendritic Cell Subsets in Health and Disease,” Immunological Reviews 219 (2007): 118–142, 10.1111/j.1600-065X.2007.00551.x. 17850486

[41] C. Wang , L. Zhong , J. Xu , et al., “Oncolytic Mineralized Bacteria as Potent Locally Administered Immunotherapeutics,” Nature Biomedical Engineering 8 (2024): 561–578, 10.1038/s41551-024-01191-w. 38514774

[42] G. Liu , N. Ma , K. Cheng , et al., “Bacteria‐Derived Nanovesicles Enhance Tumour Vaccination by Trained Immunity,” Nature Nanotechnology 19 (2024): 387–398, 10.1038/s41565-023-01553-6. 38052943

[43] J. Xu , Y. Yu , Y. Zhang , et al., “Oral Administration of Garlic‐Derived Nanoparticles Improves Cancer Immunotherapy by Inducing Intestinal IFNγ‐Producing γδ T Cells,” Nature Nanotechnology 19 (2024): 1569–1578, 10.1038/s41565-024-01722-1. 39054386

[44] A. M. van der Leun , D. S. Thommen , and T. N. Schumacher , “CD^8+^ T Cell States in Human Cancer: Insights From Single‐Cell Analysis,” Nature Reviews Cancer 20 (2020): 218–232. 32024970 10.1038/s41568-019-0235-4 PMC7115982

[45] A. Baessler and D. A. A. Vignali , “T Cell Exhaustion,” Nature Immunology 12 (2011): 492–499. 21739672 10.1038/ni.2035

[46] E. M. Janssen , E. E. Lemmens , T. Wolfe , U. Christen , M. G. von Herrath , and S. P. Schoenberger , “CD^4+^ T Cells Are Required for Secondary Expansion and Memory in CD^8+^ T Lymphocytes,” Nature 421 (2003): 852–856, 10.1038/nature01441. 12594515

[47] D. J. Shedlock and H. Shen , “Requirement for CD4 T Cell Help in Generating Functional CD8 T Cell Memory,” Science 300 (2003): 337–339, 10.1126/science.1082305. 12690201

[48] L. Yuan , B. Xu , P. Yuan , et al., “Tumor‐Infiltrating CD^4+^ T Cells in Patients With Gastric Cancer,” Cancer Cell International 17 (2017): 114, 10.1186/s12935-017-0489-4. 29213216 PMC5712164

[49] P. Fluxá , D. Rojas‐Sepúlveda , M. A. Gleisner , et al., “High CD^8+^ And Absence Of Foxp^3+^ T Lymphocytes Infiltration In Gallbladder Tumors Correlate With Prolonged Patients Survival,” BMC cancer 18 (2018): 243, 10.1186/s12885-018-4147-6. 29499656 PMC5833069

[50] Y. Huang , C. Ma , Q. Zhang , et al., “CD^4+^ and CD^8+^ T Cells Have Opposing Roles in Breast Cancer Progression and Outcome,” Oncotarget 6 (2015): 17462–17478, 10.18632/oncotarget.3958. 25968569 PMC4627321

[51] H. S. Venkatesh , L. T. Tam , P. J. Woo , et al., “Targeting Neuronal Activity‐Regulated Neuroligin‐3 Dependency in High‐Grade Glioma,” Nature 549 (2017): 533–537, 10.1038/nature24014. 28959975 PMC5891832

[52] R. Mancusi and M. Monje , “The Neuroscience of Cancer,” Nature 618 (2023): 467–479, 10.1038/s41586-023-05968-y. 37316719 PMC11146751

[53] S. Krishna , A. Choudhury , M. B. Keough , et al., “Glioblastoma Remodelling of Human Neural Circuits Decreases Survival,” Nature 617 (2023): 599–607, 10.1038/s41586-023-06036-1. 37138086 PMC10191851

[54] S. K. Tetzlaff , E. Reyhan , N. Layer , et al., “Characterizing and Targeting Glioblastoma Neuron‐Tumor Networks With Retrograde Tracing,” Cell 188 (2025): 390–411, 10.1016/j.cell.2024.11.002. 39644898

[55] Y. Zhang , W. Duan , L. Chen , et al., “Potassium Ion Channel Modulation at Cancer‐Neural Interface Enhances Neuronal Excitability in Epileptogenic Glioblastoma Multiforme,” Neuron 113 (2025): 225–243, 10.1016/j.neuron.2024.10.016. 39532103

[56] C. S. Hoyt , “Brain Injury and the Eye,” Eye 21 (2007): 1285–1289, 10.1038/sj.eye.6702849. 17914431

[57] E. P. W. Jenkins , A. Finch , M. Gerigk , I. F. Triantis , C. Watts , and G. G. Malliaras , “Electrotherapies for Glioblastoma,” Advanced Science 8 (2021): 2100978, 10.1002/advs.202100978. 34292672 PMC8456216

[58] Y. Liu , F. Zhou , H. Ali , J. D. Lathia , and P. Chen , “Immunotherapy for Glioblastoma: Current State, Challenges, and Future Perspectives,” Cellular & Molecular Immunology 21 (2024): 1354–1375, 10.1038/s41423-024-01226-x. 39406966 PMC11607068

[59] E. Song , T. Mao , H. Dong , et al., “VEGF‐C‐Driven Lymphatic Drainage Enables Immunosurveillance of Brain Tumours,” Nature 577 (2020): 689–694, 10.1038/s41586-019-1912-x. 31942068 PMC7100608

[60] W. H. Hicks , C. E. Bird , J. I. Traylor , et al., “Contemporary Mouse Models in Glioma Research,” Cells 10 (2021): 712, 10.3390/cells10030712. 33806933 PMC8004772

[61] A. Dobin , C. A. Davis , F. Schlesinger , et al., “STAR: Ultrafast Universal RNA‐Seq Aligner,” Bioinformatics 29 (2013): 15–21, 10.1093/bioinformatics/bts635. 23104886 PMC3530905

