# Supplementary material for: Eye‐Brain Neuroimmune Axis Enables Long‐Term Survival in Glioblastoma by Modulating Brain Immune Surveillance and Neuronal Excitability
Source: Adv Sci (Weinh). 2026 Jun 22;13(52):e76265. doi: 10.1002/advs.76265 (PMC13336885; doi:10.1002/advs.76265)
Supplement: Supplementary file 1 [file ADVS-13-e76265-s001.docx]

**Supplementary information**

**Eye-brain neuroimmune axis enables long-term survival in glioblastoma by modulating brain immune surveillance and neuronal excitability**

Mingyue Cui, ^1,2, †^*^,^* ^*^ Lulu Qian, ^1,2, †,^ Binbin Chu,^2, †,^ Xuan Qin,^2^ Menglin Wu,^1^ Minke Wu,^2^ Tongyu He,^2^ Baochuan Zhang,^2^ Bin Song,^2^ Yao He^1, 2 *^

^1^Macao Translational Medicine Center, Macau University of Science and Technology, Taipa 999078, Macau SAR, China

^2^Suzhou Key Laboratory of Nanotechnology and Biomedicine, Collaborative Innovation Center of Suzhou Nano Science and Technology (NANO-CIC), Soochow University, Suzhou 215123, China

* Corresponding authors. E-mail: mingyuecui@suda.edu.cn

E-mail: yaohe@suda.edu.cn/yaohe@must.edu.mo

^†^ These authors contributed equally to this work.

**Inventory of Supporting Information:**

Supplementary Figures S1-S41

Supplementary Tables S1-S2

**Table of Contents**

[Supplementary Methods	6](#_Toc230013547)

[Cells	6](#_Toc230013548)

[Isolation of mononuclear cells for flow cytometry	6](#_Toc230013549)

[Bulk RNA-seq and analysis	7](#_Toc230013550)

[EdU assay	8](#_Toc230013551)

[Behavioral assessment	8](#_Toc230013552)

[dCLN blockade and tracer drainage imaging	10](#_Toc230013553)

[dCLN dendritic-cell flow cytometry	10](#_Toc230013554)

[Live-cell Ca²⁺ imaging	11](#_Toc230013555)

[Statistical analysis	12](#_Toc230013556)

[Supplementary Figures	14](#_Toc230013557)

[Figure S1. Dye was IVT injected into the left eye with/without ES.	14](#_Toc230013558)

[Figure S2. UMAP projections of DC cell subclusters colored by expression levels of the indicated DC markers.	15](#_Toc230013559)

[Figure S3. dCLN blockade abolishes ES-enhanced antigen drainage.	16](#_Toc230013560)

[Figure S4. dCLN blockade attenuates ES-enhanced dendritic-cell maturation in deep cervical lymph nodes.	18](#_Toc230013561)

[Figure S5. Temporal immune profiling links posterior ocular antigen drainage to dCLN DC maturation and downstream T-cell involvement.	20](#_Toc230013562)

[Figure S6. Differential gene expression and pathway enrichment analysis in IVT-treated mice compared to controls.	22](#_Toc230013563)

[Figure S7. Differential gene expression and pathway enrichment analysis in IVT+ES-treated mice compared to IVT-treated mice.	23](#_Toc230013564)

[Figure S8. KEGG pathway and GO enrichment analyses of differentially expressed genes in ES-treated mice compared with controls.	24](#_Toc230013565)

[Figure S9. Differential gene expression and pathway enrichment analysis IVT+ES-treated mice compared to ES-treated mice.	25](#_Toc230013566)

[Figure S10. Differential gene expression and pathway enrichment analysis in IVT+ES-treated mice compared to controls.	27](#_Toc230013567)

[Figure S11. UMAP visualization of the T cell population, revealing multiple subclusters based on well-defined T cell markers (left panel).	28](#_Toc230013568)

[Figure S12. Pseudotime analysis of single-cell RNA-seq data from T cells in control (a) and IVT+ES (b) groups.	29](#_Toc230013569)

[Figure S13. Representative flow cytometry gating strategy for T‐cell subset analysis.	30](#_Toc230013570)

[Figure S14. Immunophenotypic analysis of tumor-infiltrating T-cell subsets in the brain tumor microenvironment.	31](#_Toc230013571)

[Figure S15. Immunophenotypic analysis of T-cell subsets in dCLNs.	32](#_Toc230013572)

[Figure S16. Immunophenotypic analysis of meningeal T-cell subsets.	33](#_Toc230013573)

[Figure S17. Immunophenotypic analysis of T-cell subsets in subclavicular lymph nodes.	34](#_Toc230013574)

[Figure S18. Representative flow cytometry gating strategy for macrophage subset analysis.	35](#_Toc230013575)

[Figure S19. Immunophenotypic analysis of tumor-infiltrating macrophage polarization.	36](#_Toc230013576)

[Figure S20. Immunophenotypic analysis of tumor-infiltrating T-cell subsets on Day 14.	37](#_Toc230013577)

[Figure S21. Quantitative analysis of CD4⁺/CD8⁺ T-cell distribution in lymphoid tissues on Day 14.	38](#_Toc230013578)

[Figure S22. Ultrastructural analysis of neuron–glioma interactions under electrical stimulation.	39](#_Toc230013579)

[Figure S23. ES attenuates neuron-driven Ca²⁺ responses in GL261 cells.	40](#_Toc230013580)

[Figure S24. Effect of electrical stimulation (ES) on GL261 glioma cell proliferation in monoculture and neuron co-culture.	42](#_Toc230013581)

[Figure S25. Effect of electrical stimulation (ES) on GL261 glioma cell viability.	43](#_Toc230013582)

[Figure S26. Electrical stimulation reduces glioma cell proliferation in neuron co-culture.	44](#_Toc230013583)

[Figure S27. Single-cell transcriptomic profiling of tumor subclusters following IVT+ES treatment.	45](#_Toc230013584)

[Figure S28. GO biological process enrichment analysis of tumor subclusters after IVT+ES treatment.	46](#_Toc230013585)

[Figure S29. Violin plots illustrating the expression patterns of marker genes for different neural cell lineages across various cell types.	47](#_Toc230013586)

[Figure S30. ES attenuates GL261-induced neuronal Ca²⁺ activity in neuron–glioma co-cultures.	48](#_Toc230013587)

[Figure S31. *In vitro* and *in vivo* safety evaluations of electrical stimulation (ES) circuit.	50](#_Toc230013588)

[Figure S32. *In vivo* safety evaluations of electrical stimulation (ES) circuit.	51](#_Toc230013589)

[Figure S33. Open-field test for evaluation of locomotor activity and anxiety-like behavior after different treatments.	52](#_Toc230013590)

[Figure S34. Rotarod test for evaluation of motor coordination and balance after different treatments.	53](#_Toc230013591)

[Figure S35. Novel object recognition (NOR) test for evaluation of recognition memory after different treatments.	54](#_Toc230013592)

[Figure S36. *In vivo* assessment of different treatments.	55](#_Toc230013593)

[Figure S37. Therapeutic efficacy of eye-brain axis electrical stimulation in intracranial GL261 GBM.	56](#_Toc230013594)

[Figure S38. *In vivo* bioluminescence imaging and survival analysis of intracranial GL261-luc GBM under different treatments.	57](#_Toc230013595)

[Figure S39. Orthotopic glioma model in nude mice.	58](#_Toc230013596)

[Figure S40. Tumor growth quantification and survival analysis in intracranial GL261 GBM mice.	59](#_Toc230013597)

[Figure S41. ES alone suppresses tumor progression and attenuates glioma-associated synaptic connectivity in GL261-bearing nude mice.	60](#_Toc230013598)

[Supplementary Tables	62](#_Toc230013599)

[Table S1. *In vivo* frequency-screening experiment used to select the working ES condition for subsequent studies.	62](#_Toc230013600)

[Table S2. The antibodies used in this study.	63](#_Toc230013601)

[Reference	65](#_Toc230013602)

# Supplementary Methods

## Cells

GL261-Luc cells, ARPE-19 cells, and HUVECs were purchased from Shanghai Zhong Qiao Xin Zhou Biotechnology Co., Ltd. (China). GL261-Luc cells were cultured in RPMI-1640 medium supplemented with 10% fetal bovine serum (FBS), 1% penicillin–streptomycin, and 1% sodium pyruvate. ARPE-19 cells and HUVECs were maintained in H-DMEM containing 10% FBS and 1% penicillin–streptomycin.

## **Isolation of mononuclear cells for flow cytometry**

For brain tumor samples, tissues were harvested and digested in a solution containing 1 mg mL⁻¹ collagenase D (Roche) and 30 μg mL⁻¹ DNase I (Sigma-Aldrich) in RPMI medium at 37 °C for 45 min. After digestion, tissues were mechanically dissociated by repeated pipetting and passed through a 70 μm cell strainer.

The resulting suspension was mixed with 3 mL of 25% Percoll (Sigma-Aldrich) and centrifuged at 580 g for 15 min without brake. After removal of the Percoll layer, cell pellets were treated with 0.5 mL ACK lysis buffer and centrifuged again at 500 g for 5 min. The final pellet was resuspended in FACS buffer (PBS containing 2% FBS and 1 mM EDTA) for staining. For lymphocyte analysis, lymph nodes (LNs) or meninges were placed in a 60 mm × 15 mm Petri dish containing 2 mL FACS buffer and gently dissociated between two frosted microscope slides. Cell suspensions were subsequently filtered through a 70 μm strainer and centrifuged at 500 g for 5 min, after which the pellets were resuspended in FACS buffer for antibody staining. Cells were incubated with appropriate antibodies for 30 min at 4 °C, washed to remove unbound antibodies, and resuspended in FACS buffer prior to analysis. Flow cytometric acquisition was performed using a BD Aria III cell sorter, and data were analyzed with FlowJo software (v10.8.1, Tree Star).

To assess immune alterations induced by eye–brain axis–mediated CNS immunization under different treatments, immune profiling was performed on tumor tissues, lymph nodes, or meninges. Briefly, mice were euthanized on days 7 and 14, tissues were collected, single-cell suspensions were prepared, and immune cell populations were analyzed by flow cytometry. The following markers were used for immune cell characterization: (1) CD3 T cells: FITC-CD3; (2) CD4 T cells: FITC-CD3 and PE-CD4; (3) CD8 T cells: FITC-CD3 and APC-CD8a; (4) M1 macrophages: FITC-CD11c and APC-CD80; (5) M2 macrophages: FITC-CD11c and APC-CD206. Cytokine levels (IFN-γ, IL-6, TNF-α, and IL-10) in culture supernatants were quantified using enzyme-linked immunosorbent assays (ELISA).

## **Bulk RNA-seq and analysis**

Total RNA was isolated from tumour tissues of control mice (n = 3) and IVT+ES–treated mice (n = 3) using TRIzol reagent (Invitrogen Life Technologies). RNA integrity was evaluated with an Agilent 2100 Bioanalyzer (Agilent Technologies, Palo Alto, CA, USA), and RNA concentration was determined using a NanoDrop ND-2000 spectrophotometer (NanoDrop Technologies, Wilmington, DE, USA).

Only RNA samples meeting the following quality criteria were used for library preparation: OD260/280 ratio of 1.8–2.2, OD260/230 ≥ 2.0, RNA integrity number (RIN) ≥ 6.5, 28S:18S ≥ 1.0, and total RNA ≥ 1 μg. RNA purification, reverse transcription, library preparation, and sequencing were carried out by Shanghai Novel Biotechnology Co., Ltd. (Shanghai, China) in accordance with Illumina standard protocols. Briefly, mRNA was enriched and fragmented, followed by synthesis of double-stranded cDNA using the SuperScript double-stranded cDNA synthesis kit (Invitrogen Life Technologies, 11917020).

The resulting cDNA libraries underwent end repair, phosphorylation, and A-tailing according to the Illumina workflow. Fragments of approximately 300 bp were selected using 2% low-range ultra-agarose gel electrophoresis, followed by 15 cycles of PCR amplification with Phusion DNA polymerase (New England Biolabs, Ipswich, MA, USA). Paired-end RNA-seq libraries were sequenced on an Illumina NovaSeq 6000 platform with a read length of 2 × 150 bp. Raw sequencing reads were processed for adapter trimming and quality filtering using fastp (v0.19.4) with default parameters. Clean reads were subsequently aligned to the mouse reference genome (mm10) using HISAT2 ^1^. Aligned reads from each sample were assembled with StringTie using a reference-guided approach. Differential gene expression analysis between groups was performed with the DESeq2 package (v3.12.1). Genes with |log₂(fold change)| > 1 and P < 0.05 were considered significantly differentially expressed ^2^.

## **EdU assay**

Cell proliferation was evaluated using an EdU incorporation assay kit (Invitrogen) following the manufacturer’s protocol. GL261 glioma cells were plated onto poly-D-lysine- and laminin-coated coverslips at a density of 1 × 10⁴ cells per well in 24-well plates. Approximately 24 h after plating, embryonic mouse hippocampal neurons were introduced at 4 × 10⁴ cells per well to establish the glioma–neuron co-culture condition.

After 72 h of culture, glioma cells grown either alone or together with neurons were exposed to 20 μM EdU overnight at 37 °C. Cells were then fixed with 4% paraformaldehyde (PFA) and processed for staining according to the Click-iT EdU kit protocol. The proliferation index was calculated by measuring the proportion of EdU-positive cells relative to total DAPI-stained nuclei using confocal microscopy at 20× magnification.

## Behavioral assessment

To evaluate whether the eye-brain electrical stimulation (ES) circuit induced potential neurological deficits, behavioral assessments were performed after the indicated treatments, including the open-field test, rotarod test, and novel object recognition test. Mice were transferred to the behavioral testing room at least 30 min before testing for acclimation. All tests were conducted under consistent environmental conditions, and the apparatus was cleaned between animals to remove olfactory cues. Behavioral data were recorded and analyzed in a blinded manner.

(1) **Open-field test**

The open-field test was performed to evaluate spontaneous locomotor activity and anxiety-like behavior. Briefly, each mouse was gently placed in the center of an open-field arena and allowed to freely explore the arena for 5 min. The movement trajectory was recorded using a video tracking system. The central zone was defined consistently for all animals, and the central distance traveled and time spent in the central zone were quantified. These parameters were used to assess general locomotor activity and anxiety-like behavior after different treatments.

(2) **Rotarod test**

The rotarod test was used to assess motor coordination and balance. Before testing, mice were trained on the rotating rod at a constant speed of 5 rpm for 3 min. During the testing phase, mice were placed on an accelerating rotarod, with the speed gradually increasing from 5 to 40 rpm over a maximum duration of 5 min. The latency to fall from the rotating rod was recorded for each mouse. Each mouse was tested in three trials with an interval between trials, and the average latency to fall was used for statistical analysis.

(3) **Novel object recognition test**

The novel object recognition test was performed to evaluate recognition memory and cognitive function. The test consisted of three phases: habituation, training, and testing. During the habituation phase, mice were allowed to freely explore an empty arena. During the training phase, two identical objects were placed in the arena, and mice were allowed to explore both objects. During the testing phase, one familiar object was replaced with a novel object, and the exploration time for the familiar and novel objects was recorded. The preference index was calculated as follows:

$$Preference index=\frac{T_{\text{novel}}}{T_{\text{familiar}}+T_{\text{novel}}}$$

where $T_{\text{novel}}$and $T_{\text{familiar}}$represent the exploration time for the novel and familiar objects, respectively.

## dCLN blockade and tracer drainage imaging

Deep cervical lymph node (dCLN) blockade was performed to functionally interrupt the posterior ocular–dCLN drainage pathway. Briefly, mice were anesthetized and placed in the supine position. After disinfection of the ventral neck region, a small midline cervical incision was made to expose the deep cervical lymph nodes under a stereomicroscope. The dCLNs were carefully identified according to their anatomical location adjacent to the trachea and carotid sheath, and were blocked by local electrocautery while leaving the superficial cervical lymph nodes (sCLNs) intact. Sham-operated mice underwent the same surgical exposure without electrocautery blockade. After surgery, mice were allowed to recover before subsequent intravitreal administration and electrical stimulation treatment.

For tracer drainage imaging, FITC-dextran was used as a fluorescent tracer to evaluate ocular lymphatic drainage. Mice received intravitreal injection of FITC-dextran into the left eye, followed by electrical stimulation where indicated. At 1 h after tracer injection, mice were euthanized, and the eyes, sCLNs, and dCLNs were collected for *ex vivo* fluorescence imaging using an IVIS imaging system. Fluorescence signals were acquired under identical imaging settings across groups and quantified as background-subtracted radiant efficiency or relative fluorescence intensity.

## dCLN dendritic-cell flow cytometry

Deep cervical lymph nodes (dCLNs) were collected at 24 h after treatment to assess early dendritic-cell activation and maturation. Briefly, mice were euthanized, and dCLNs were carefully dissected under a stereomicroscope. The collected dCLNs were placed in cold FACS buffer consisting of PBS supplemented with 2% fetal bovine serum and 1 mM EDTA. Single-cell suspensions were prepared by gently dissociating the lymph nodes between two frosted glass slides, followed by filtration through a 70 μm cell strainer. The cell suspensions were centrifuged at 500 g for 5 min at 4 °C, and the pellets were resuspended in FACS buffer. For surface staining, cells were incubated with Fc-blocking reagent for 10 min at 4 °C to minimize nonspecific antibody binding, followed by staining with fluorophore-conjugated antibodies against CD11c, MHC-II, CD80, and CD86 for 30 min at 4 °C in the dark. After staining, cells were washed twice with FACS buffer and resuspended in FACS buffer for flow-cytometric acquisition. Flow cytometry was performed using a BD Aria III cell sorter, and data were analyzed using FlowJo software. For gating analysis, lymphocytes were first selected according to forward- and side-scatter properties, followed by singlet gating. Dendritic cells were identified as CD11c⁺MHC-II⁺ cells. DC maturation was further evaluated by quantifying CD80⁺CD86⁺ cells within the CD11c⁺MHC-II⁺ DC population. The percentages of CD11c⁺MHC-II⁺ DCs among lymphocyte singlets and CD80⁺CD86⁺ mature DCs among CD11c⁺MHC-II⁺ DCs were used to compare dendritic-cell activation among the indicated groups.

## Live-cell Ca²⁺ imaging

Live-cell Ca²⁺ imaging was performed using Fluo-4 AM to monitor intracellular Ca²⁺ dynamics in GL261 cells and primary hippocampal neurons. Briefly, cells were washed with pre-warmed calcium imaging buffer and incubated with 5 μM Fluo-4 AM containing 0.02% Pluronic F-127 at 37 °C for 30 min in the dark. After dye loading, cells were washed three times with calcium imaging buffer and incubated for an additional 15 min to allow de-esterification of Fluo-4 AM. Cells were then maintained in calcium imaging buffer during imaging. Fluorescence images were acquired using a confocal microscope with excitation at 488 nm, and time-lapse images were recorded under identical imaging settings across all groups. Intracellular Ca²⁺ responses were quantified as ΔF/F₀, where F₀ represents the baseline fluorescence intensity and ΔF represents the change in fluorescence intensity relative to baseline.

**(1) GL261 Ca²⁺ imaging**

GL261-Luc cells were cultured either alone or co-cultured with primary hippocampal neurons to evaluate neuron-associated Ca²⁺ activity in glioma cells. For co-culture experiments, GL261-Luc cells were pre-labeled with CellTracker™ Deep Red Dye according to the manufacturer’s instructions to distinguish GL261 cells from neurons. After labeling, GL261-Luc cells were co-cultured with primary hippocampal neurons and subjected to the indicated treatments.

After Fluo-4 AM loading, live-cell Ca²⁺ imaging was performed using confocal microscopy. In neuron–GL261 co-cultures, Ca²⁺ signals were quantified specifically in CellTracker™ Deep Red-positive GL261 cells. In selected groups, CNQX (10 μM) and APV (50 μM) were added before imaging to inhibit AMPA/kainate receptor- and NMDA receptor-mediated glutamatergic signaling, respectively. Time-lapse Fluo-4 fluorescence signals were recorded, and the peak Ca²⁺ response in GL261 cells was calculated as peak ΔF/F₀. This assay was used to assess whether neuronal co-culture enhanced GL261 Ca²⁺ activity and whether ES treatment attenuated neuron-associated glioma Ca²⁺ responses.

**(2) Neuron Ca²⁺ imaging**

Primary hippocampal neurons were used to evaluate ES-associated modulation of neuronal excitability. After the indicated treatments, neurons were loaded with Fluo-4 AM as described above and maintained in calcium imaging buffer for live-cell imaging. Baseline and spontaneous Ca²⁺ activity were recorded by time-lapse confocal microscopy under identical imaging settings across all groups. In selected control groups, TTX (1 μM) was added before imaging to block voltage-gated Na⁺ channel-dependent action potential generation and suppress neuronal firing-associated Ca²⁺ activity. Regions of interest (ROI) were manually drawn around individual neuronal somata, and fluorescence intensity changes were measured over time. Neuronal Ca²⁺ activity was quantified as peak ΔF/F₀ and/or mean ΔF/F₀ during the recording period. Reduced spontaneous Ca²⁺ activity after ES treatment, together with the inhibitory effect of TTX, was used as functional evidence supporting ES-associated attenuation of neuronal excitability.

## Statistical analysis

Statistical analyses were performed using GraphPad Prism software. Comparisons between two groups were evaluated using a two-tailed unpaired Student’s t-test, whereas one-way ANOVA followed by Tukey’s multiple-comparisons test was applied for analyses involving multiple groups. Statistical significance was defined as **P < 0.05, **P < 0.01, and ***P < 0.001*.

# Supplementary Figures

Figure S1. Dye was IVT injected into the left eye with/without ES. Eyes, sCLNs and dCLNs were collected for IVIS epifluorescence imaging. Representative background-subtracted heat maps of dye in the eye, sCLNs and dCLNs 1 h later after injection.

Figure S2. UMAP projections of DC cell subclusters colored by expression levels of the indicated DC markers. cDC1 subclusters are identified by high XCR1, CLEC9A, and IRF8 expression, cDC2 subclusters by high CD11b and SIRPα, and pDC subclusters by high CD317 and Siglec-H. Each marker’s expression intensity is represented by the color scale on the right^3-4^.

Figure S3. dCLN blockade abolishes ES-enhanced antigen drainage. (a) Schematic of the experimental workflow. (1) Surgical blockade of the deep cervical lymph nodes (dCLN) using electrocautery, leaving the superficial cervical lymph nodes (sCLN) intact. (2) Intravitreal (IVT) injection of FITC tracer. (3) Tracer drainage pathway under sham versus dCLN-blocked conditions. (4) Representative fluorescence readout showing high tracer signal in dCLN for sham mice and markedly reduced signal when dCLN is blocked. (**b)** Representative FITC distribution in eye, sCLN, and dCLN across groups: control, IVT, IVT + ES, IVT + ES + Sham, IVT + ES + dCLN blockade. **(c–e)** Quantification of tracer fluorescence in eye (**c**), sCLN (**d**), and dCLN (**e**). Data are mean ± SD (n = 3). Statistical significance was analyzed using one-way ANOVA followed by Tukey’s multiple-comparisons test. *p* < 0.05, ****p* < 0.0001; ns, not significant.

We surgically blocked the posterior ocular–dCLN drainage route before intravitreal administration and ES treatment. As illustrated in Figure S3, dCLN blockade markedly reduced ES-enhanced tracer accumulation in dCLNs, indicating that ES-mediated ocular antigen transport depends on an intact posterior ocular–dCLN drainage pathway.

Figure S4. dCLN blockade attenuates ES-enhanced dendritic-cell maturation in deep cervical lymph nodes. **(a)** Schematic illustration of the experimental timeline. **(b)** Representative gating strategy for flow-cytometric analysis of dCLN dendritic cells. **(c)** Quantification of CD11c⁺MHC-II⁺ DCs among lymphocyte singlets in dCLNs from different treatment groups. **(d)** Representative flow-cytometry plots showing CD80 and CD86 expression in CD11c⁺MHC-II⁺ DCs. **(e)** Quantification of CD80⁺CD86⁺ mature DCs among CD11c⁺MHC-II⁺ DCs. Data are presented as mean ± SD. n=3. Statistical significance was analyzed using one-way ANOVA followed by Tukey’s multiple-comparisons test. ****P < 0.001, ****P < 0.0001.*

We examined whether disruption of this pathway attenuated downstream immune activation. As shown in Figure S4, IVT+ES markedly increased the proportion of CD80⁺CD86⁺ mature DCs within the CD11c⁺MHC-II⁺ population to ~41%, compared with ~9.7% in the control group and ~25% in the IVT group. This DC maturation pattern was preserved in the sham-operated group but was reduced to ~14% after dCLN blockade. These results indicate that the intact posterior ocular–dCLN route is required for ES-enhanced DC maturation and antigen-presentation-associated immune priming in dCLNs. Together with the tracer-based drainage data, these findings functionally support the involvement of the posterior ocular–dCLN pathway in ES-enhanced antigen transport and DC maturation.

Figure S5. Temporal immune profiling links posterior ocular antigen drainage to dCLN DC maturation and downstream T-cell involvement. **(a)** Schematic illustration of the experimental timeline and proposed immune sequence. GL261-bearing mice received IVT and/or ES treatment on Days 1 and 5. dCLNs were harvested at 24 h after treatment for DC analysis and at 48 h after treatment for T-cell profiling. **(b)** Quantification of CD11c⁺MHC-II⁺ DCs among lymphocyte singlets in dCLNs. **(c)** Quantification of CD80⁺CD86⁺ mature DCs among CD11c⁺MHC-II⁺ DCs in dCLNs. **(d)** Quantification of the dCLN CD4⁺/CD8⁺ T-cell ratio at 48 h after treatment. Data are presented as mean ± SD. n=3. Statistical significance was determined by one-way ANOVA with multiple comparisons. **P < 0.05, **P < 0.01, ***P < 0.001, ****P < 0.0001.*

As shown in Figure S5a, dCLNs were harvested at 24 h after treatment for DC analysis, whereas dCLN T-cell profiling was performed at 48 h after treatment. At 24 h, IVT+ES increased the proportion of CD11c⁺MHC-II⁺ DCs among lymphocyte singlets and markedly enhanced CD80⁺CD86⁺ mature DCs within the CD11c⁺MHC-II⁺ DC population. In particular, CD80⁺CD86⁺ mature DCs increased from ~25% in the IVT group to ~41% in the IVT+ES group, indicating enhanced antigen-presentation-associated DC maturation in dCLNs. We further connected this early DC-maturation event with downstream T-cell profiling. In Figure S5d, IVT+ES increased the dCLN CD4⁺/CD8⁺ T-cell ratio at 48 h compared with IVT alone, supporting subsequent T-cell profile changes following DC maturation. Together, these data support a temporal immune sequence from posterior ocular antigen drainage to dCLN DC maturation and subsequent T-cell involvement.

Figure S6. Differential gene expression and pathway enrichment analysis in IVT-treated mice compared to controls. (a) Volcano plot of differentially expressed genes in IVT-treated mice compared to controls (n=3 mice). Genes meeting the threshold of fold change (FC) > 1.5 and false discovery rate (FDR) < 0.05 are highlighted in red or blue. (b) KEGG pathway enrichment analysis of the differentially expressed genes. Aberrant activation of the PI3K-Akt signaling pathway is a common feature in a wide range of cancers, particularly those characterized by enhanced cellular proliferation, evasion of apoptosis, and increased invasiveness and metastatic potential. The bar length indicates the relative statistical significance of each pathway, with longer bars reflecting lower P-values.

Figure S7. Differential gene expression and pathway enrichment analysis in IVT+ES-treated mice compared to IVT-treated mice. (a) Volcano plot of differentially expressed genes in IVT+ES-treated mice compared to IVT-treated mice (n=3 mice). Genes meeting the threshold of fold change (FC) > 1.5 and false discovery rate (FDR) < 0.05 are highlighted in red. The top significantly changed genes are labeled. (b) KEGG pathway enrichment analysis of the differentially expressed genes. The top enriched pathways, including IL-17 signaling pathway, TNF signaling pathway, and NF-κ signaling pathway, etc. The bar length indicates the relative statistical significance of each pathway, with longer bars reflecting lower P-values. Notably, the combination of IVT and ES (IVT+ES) treatment was found to activate pro-apoptotic pathways, underscoring a potential mechanism for tumor suppression.

Figure S8. KEGG pathway and GO enrichment analyses of differentially expressed genes in ES-treated mice compared with controls. (a) GO Biological Process (BP) enrichment analysis of upregulated genes, displaying the top significantly enriched BP terms. (b) GO Cellular Component (CC) enrichment analysis of downregulated genes, similarly depicting the most significantly enriched CC terms.

Electrical stimulation has been shown to attenuate neuronal excitability, reduce synapse formation, and thereby inhibit the proliferative capacity of tumor cells.

Figure S9. Differential gene expression and pathway enrichment analysis IVT+ES-treated mice compared to ES-treated mice. (a) Volcano plot of differentially expressed genes in IVT+ES-treated mice compared to ES- treated mice (n=3 mice). Genes meeting the threshold of fold change (FC) > 1.5 and false discovery rate (FDR) < 0.05 are highlighted in red or blue. The top significantly changed gene Cldn4 is highlighted by a red circle. **(b)** KEGG pathway enrichment analysis of the top 20 enriched pathways (down regulation). Each bubble represents an enriched pathway, with bubble size indicating the number of genes and color denoting the level of statistical significance (p-value).

In tumor tissues, elevated expression of Cldn4 is significantly correlated with the loss of cellular polarity, disruption of tight junction integrity, and an increase in both cellular proliferation and metastatic potential. Moreover, experimental inhibition of Cldn4 expression has been demonstrated to effectively suppress the growth of brain tumor cells.

Figure S10. Differential gene expression and pathway enrichment analysis in IVT+ES-treated mice compared to controls. (a) Volcano plot of differentially expressed genes in IVT+ES-treated mice compared to controls. Genes meeting the threshold of fold change (FC) > 1.5 and false discovery rate (FDR) < 0.05 are highlighted in red (upregulated, n=175) or blue (downregulated, n=87). The top significantly changed genes are labeled. (b) KEGG pathway enrichment analysis of the differentially expressed genes. Each bubble represents an enriched pathway, with bubble size indicating the number of genes and color denoting the level of statistical significance (p-value).

Figure S11. UMAP visualization of the T cell population, revealing multiple subclusters based on well-defined T cell markers (left panel). These subclusters include naive T cells (*Tcf7*), exhausted CD8^+^ T (CD8^+^ Tex) cells (*Cd8a, Tim3*), cytotoxic CD8^+^ T (CD8^+^ Teff) cells (*Cd8a*, *Gzmk*), CD4^+^ Th1 cells (*Cd4*, *Ifng*), CD4^+^ Th10 cells (*Cd4*, *IL10*) and regulatory CD4^+^ T (CD4^+^ Treg) cells (*Foxp3*). Each subcluster is annotated according to its phenotypic identity (e.g., CD4^+^T-Naive, CD4^+^T-Treg, T-Cycling). The right panel shows the expression patterns of representative T cell–associated genes (*Cd4*, *Cd8a*, *Tim3*, *Stmn1*, *Foxp3*, *Gzmk*, *Tcf7*, *Ifng*, *Il10*, and *Tnf*), with color intensity reflecting expression levels in each subcluster.

Figure S12. Pseudotime analysis of single-cell RNA-seq data from T cells in control (a) and IVT+ES (b) groups. Each point represents a single T cell, colored by its assigned subset identity (CD8^+^ Tex, CD4^+^ Th1, CD8^+^ Teff, Naive T, CD4^+^ Tcm, CD4^+^ Treg, and CD4^+^ Th10). Branch points (Fate 1, Fate 2) mark key differentiation pathways along the trajectory. The results revealed that the proportions of CD8^+^ Teff cells increased along the trajectory in mice receiving IVT+ES immunization.

Figure S13. Representative flow cytometry gating strategy for T‐cell subset analysis. T cells (CD3⁺) were identified using FITC‐conjugated anti‐CD3, followed by further delineation into CD4⁺ and CD8⁺ subsets using APC‐conjugated anti‐CD4 and anti‐CD8, respectively^5-6^.

Figure S14. Immunophenotypic analysis of tumor-infiltrating T-cell subsets in the brain tumor microenvironment. Flow cytometric analysis of tumor‐infiltrating lymphocytes (TILs) in a mouse brain tumor model under different treatment conditions (Control, anti-PD1, IVT, ES, anti-PD1+IVT, IVT+ES) (n=3 mice). The left panel of each treatment shows the percentage of CD4⁺ T cells, while the right panel shows the percentage of CD8⁺ T cells.

T cell analysis in the TME demonstrated that the CD4^+^/CD8^+^ T cell ratios in the IVT+anti-PD1 and IVT+ES groups were both less than 1, with the IVT+ES group showing an even lower ratio. CD8^+^ T cells were significantly upregulated in the IVT+ES group compared to CD4^+^ T cells and other groups. These findings suggest that IVT+ES shifts T‐cell balance toward a higher proportion of CD8⁺ T cells, potentially enhancing anti‐tumor immunity.

Figure S15. Immunophenotypic analysis of T-cell subsets in dCLNs. Flow cytometric analysis of T‐cell populations in the draining cervical lymph nodes (dCLNs) under different treatment conditions (Control, anti-PD1, IVT, ES, anti-PD1+IVT, IVT+ES) (n=3 mice). The left panel of each treatment shows the percentage of CD4⁺ T cells, while the right panel shows the percentage of CD8⁺ T cells.

Combining IVT with ES significantly elevates the CD4⁺/CD8⁺ ratio in dCLNs, suggesting an enhanced helper T‐cell response.

Figure S16. Immunophenotypic analysis of meningeal T-cell subsets. Flow cytometric analysis of T‐cell populations in the meninges under different treatment conditions (Control, anti-PD1, IVT, ES, anti-PD1+IVT, IVT+ES) (n=3 mice). The left panel of each treatment shows the percentage of CD4⁺ T cells, while the right panel shows the percentage of CD8⁺ T cells. The CD4⁺/CD8⁺ ratio (in red) is highest in the IVT+ES group (1.55), indicating a pronounced increase in CD4⁺ T‐cell infiltration.

Figure S17. Immunophenotypic analysis of T-cell subsets in subclavicular lymph nodes. Flow cytometric analysis of T‐cell populations in the subclavicular lymph nodes (sCLN) under different treatment conditions (Control, anti-PD1, IVT, ES, anti-PD1+IVT, IVT+ES) (n=3 mice). The left panel of each treatment shows the percentage of CD4⁺ T cells, while the right panel shows the percentage of CD8⁺ T cells.

Conversely, in the sCLNs, the CD4^+^/CD8^+^ T cell ratio was highest in the anti-PD1 group, likely due to the systemic immune effects of peritoneally injected anti-PD1, which promoted CD4^+^ T cell proliferation and activation.

Figure S18. Representative flow cytometry gating strategy for macrophage subset analysis. Lymphocytes were initially gated by forward scatter (FSC‐A) versus side scatter (SSC‐A), followed by selection of singlet cells based on FSC‐H versus FSC‐A. Macrophages were identified using PE‐CD11b and FITC‐F4/80, and subsequently characterized as M1‐like (CD80⁺) or M2‐like (CD206⁺) subsets^7-8^.

Figure S19. Immunophenotypic analysis of tumor-infiltrating macrophage polarization. Flow cytometric analysis of tumor‐infiltrating macrophages (TIMs) in a mouse brain tumor model under different treatment conditions (Control, anti-PD1, IVT, ES, anti-PD1+IVT, IVT+ES) (n=3 mice). M1‐like (CD80⁺) macrophages are shown in the left panel, and M2‐like (CD206⁺) macrophages in the right panel, with the M1/M2 ratio (in red) noted above each pair of plots.

IVT+ES treatment significantly increased the proportion of M1 macrophages, contributing to the reshaping of the TME.

Figure S20. Immunophenotypic analysis of tumor-infiltrating T-cell subsets on Day 14. Flow cytometric analysis of tumor‐infiltrating CD4⁺ and CD8⁺ T cells in a mouse brain tumor model on Day 14 under different treatment conditions (n=3 mice). The left panel in each treatment shows the percentage of CD4⁺ T cells, and the right panel shows the percentage of CD8⁺ T cells.

On day 14, the proportion of CD8^+^ T cells increased across all groups, with a more pronounced elevation observed in the ES, IVT+anti-PD1, and IVT+ES groups.

Figure S21. Quantitative analysis of CD4⁺/CD8⁺ T-cell distribution in lymphoid tissues on Day 14. Statistical data showing CD4^+^/CD8^+^ T cells in dCLNs, meninges as well as sCLNs induced by different treatments *in vivo* on day 14 (n = 3 mice).

Similar to the trend recorded on day 7, the proportion of CD4^+^ T cells in the dCLNs and meninges increased on day 14, indicating sustained immune activation.

Figure S22. Ultrastructural analysis of neuron–glioma interactions under electrical stimulation. Scanning electron microscopy (SEM) was performed on glioma cells co-cultured with mouse hippocampal neurons to visualize morphological changes under either electrical stimulation (ES) or non-stimulation conditions. (a) Schematic representation (top) and SEM image (bottom) showing the co-culture before ES, with neurons (pink) forming multiple contacts (red arrows) with glioma cells (red). (b) After ES, notable alterations in neuronal processes and reduced clustering of glioma cells are observed, indicating that electrical stimulation modulates neuron–glioma interactions.

Figure S23. ES attenuates neuron-driven Ca²⁺ responses in GL261 cells. **(a)** Representative Fluo-4 AM fluorescence images of GL261 cells cultured alone with or without ES treatment. **(b)** Representative confocal images of neuron–GL261 co-cultures after Fluo-4 AM loading. GL261 cells were pre-labeled with CellTracker™ Deep Red Dye to distinguish them from primary hippocampal neurons. The merged images show Fluo-4 AM Ca²⁺ signals and CellTracker™ Deep Red-positive GL261 cells under different conditions. Scale bars, 50 μm. **(c)** Quantification of Ca²⁺ responses in CellTracker™ Deep Red-positive GL261 cells, expressed as peak ΔF/F₀. Data are presented as mean ± SD. n=5. CNQX/APV, pharmacological blockers of AMPA/kainate and NMDA receptor-mediated glutamatergic signaling. Statistical significance was analyzed using one-way ANOVA followed by Tukey’s multiple-comparisons test. NS, not significant; *****P < 0.0001*.

Specifically, GL261-Luc cells were pre-labeled with CellTracker™ Deep Red Dye to distinguish them from primary hippocampal neurons, and Fluo-4 AM was used to monitor intracellular Ca²⁺ dynamics. As illustrated in Figure S23, ES treatment alone did not significantly alter Ca²⁺ responses in GL261 monoculture, indicating no obvious direct effect of ES on basal GL261 Ca²⁺ activity. However, co-culture with neurons markedly enhanced Ca²⁺ responses in GL261 cells, whereas ES significantly reduced this neuron-driven response. CNQX/APV treatment produced a similar reduction, supporting the involvement of AMPA/kainate and NMDA receptor-mediated glutamatergic signaling. These results suggest that ES weakens neuron-driven GL261 Ca²⁺ activity, partly through attenuation of glutamatergic neuron-to-glioma communication.

Figure S24. Effect of electrical stimulation (ES) on GL261 glioma cell proliferation in monoculture and neuron co-culture. (a) Representative fluorescence images of GL261 cells (DAPI, blue; EdU, green) under the indicated conditions: ES− (no stimulation) and ES+ (electrical stimulation). Scale bar, 50 µm. (b) Quantification of the proliferation index, defined as the fraction of DAPI-positive cells co-expressing EdU. Data are presented as mean ± s.d. (n = 5). Statistical significance was analyzed using an unpaired two-tailed Student’s t-test. NS, not significant.

Figure S25. Effect of electrical stimulation (ES) on GL261 glioma cell viability. CCK-8 analysis of GL261 cells cultured alone with or without ES treatment. Data are presented as mean ± s.d. (n = 6). Statistical significance was analyzed using an unpaired two-tailed Student’s t-test. NS, not significant.

CCK-8 analysis showed no significant reduction in GL261 cell viability after ES treatment.

Figure S26. Electrical stimulation reduces glioma cell proliferation in neuron co-culture. (a) Representative fluorescence images of GL261 glioma cells co-cultured with primary neurons in the absence or presence of electrical stimulation (ES). EdU-positive proliferating cells are shown in green, and nuclei are stained with DAPI (blue). Scale bars, 50 μm. (b) Quantification of the proliferation index of GL261 cells under neuron co-culture conditions with or without ES. Data are presented as mean ± s.d. (n = 5)**.** Statistical significance was analyzed using one-way ANOVA followed by Dunnett’s multiple-comparisons test. ****P < 0.001.*

Figure S27. Single-cell transcriptomic profiling of tumor subclusters following IVT+ES treatment. IVT+ES treatment reshapes the tumor landscape by reducing proliferative and immunosuppressive cell populations while promoting immune activation. (a) UMAP plots of tumor cells in the Control and IVT+ES groups, generated through unsupervised clustering to identify distinct subclusters. (b) Bar chart showing the proportion of each subcluster in Control versus IVT+ES samples. The IVT+ES group exhibits a marked reduction in clusters 0, 1, and 2 (associated with enhanced proliferation and immunosuppressive features) and a relative enrichment in clusters 3, 4, and 5 (characterized by upregulated immune-related pathways).

Figure S28. GO biological process enrichment analysis of tumor subclusters after IVT+ES treatment. Gene Ontology (GO) enrichment analysis of biological processes (BP) in each subcluster, illustrating the shift from proliferative and immunosuppressive functions in Control to immune-activated pathways—such as immune response and antigen processing—in IVT+ES. Collectively, these findings indicate that IVT+ES treatment reprograms the intratumoral cell population from a proliferative, immunosuppressive state to an immune-activated state, potentially contributing to improved therapeutic outcomes.

Figure S29. Violin plots illustrating the expression patterns of marker genes for different neural cell lineages across various cell types. (a) Oligodendrocyte precursor cell (OPC) and oligodendrocyte markers, including Sox10, CNPase, Olig1, Olig2, PLP1, and PDGFRα, showing specific enrichment in OPCs and oligodendrocytes. (b) Neural progenitor cell (NPC) markers, including Sox4, Sox11, DCX, and STMN2, exhibiting preferential expression in NPCs. The data indicate distinct lineage-specific expression profiles across different brain cell populations.

Figure S30. ES attenuates GL261-induced neuronal Ca²⁺ activity in neuron–glioma co-cultures. **(a)** Representative Fluo-4 AM fluorescence images of primary hippocampal neurons cultured alone with or without ES treatment. **(b)** Representative confocal images of neuron–GL261 co-cultures after Fluo-4 AM loading. GL261 cells were pre-labeled with CellTracker™ Deep Red Dye to distinguish them from neurons. Green, Fluo-4 AM Ca²⁺ signal; red, CellTracker™ Deep Red-labeled GL261 cells. TTX, tetrodotoxin, was used as a voltage-gated Na⁺ channel blocker to suppress action potential-associated neuronal activity. Scale bars, 50 μm. **(c)** Quantification of peak ΔF/F₀ in neurons. Data are presented as mean ± SD. n=5. Statistical significance was analyzed using one-way ANOVA followed by multiple-comparisons test. NS, not significant; **P < 0.05, **P < 0.01.*

Specifically, primary hippocampal neurons were cultured alone or co-cultured with GL261 cells, followed by ES treatment and real-time Ca²⁺ imaging. As illustrated in Figure S30, ES reduced neuronal Ca²⁺ responses in neuron monoculture and also attenuated the elevated neuronal Ca²⁺ activity induced by GL261 co-culture. TTX, a voltage-gated Na⁺ channel blocker that suppresses action potential generation, produced a similar reduction in neuronal Ca²⁺ responses. These data provide functional evidence that ES attenuates neuronal Ca²⁺ activity and support the proposed reduction of neuronal excitability in Figure 4Q.

Figure S31. *In vitro* and *in vivo* safety evaluations of electrical stimulation (ES) circuit. (a) Schematic of the *in vitro* setup, showing the electrical generator connected to stimulating electrodes and an oscilloscope (200 kHz). (b) Representative voltage trace recorded during electrical stimulation. (c, d) Viability of ARPE (c) and HUVEC (d) cells under untreated and ES conditions (n=6, mean ± s.d.).

Figure S32. *In vivo* safety evaluations of electrical stimulation (ES) circuit. Representative H&E-stained ocular cross-sections from different treatment groups (Control, ES, anti-PD1, IVT+ anti-PD1, IVT, and IVT+ES). Scale bars: 500 µm.

Figure S33. Open-field test for evaluation of locomotor activity and anxiety-like behavior after different treatments. **(a)** Representative movement traces of mice in the open-field arena from the indicated groups. **(b)** Quantification of central distance traveled in the open-field test. **(c)** Quantification of central time spent in the central zone. Data are presented as mean ± s.d. (n = 3 mice per group). Statistical analysis was performed using one-way ANOVA followed by Tukey’s multiple-comparisons test. No significant differences were observed among groups.

Figure S34. Rotarod test for evaluation of motor coordination and balance after different treatments. (a) Schematic illustration of the rotarod test procedure. Mice were first trained on the rotarod at a constant speed (5 rpm) for 3 min, followed by the testing phase under accelerating speed conditions (5–40 rpm) for up to 5 min. The latency to fall was recorded as an index of motor coordination and balance. (b) Quantification of latency to fall in the indicated groups. Data are presented as mean ± s.d. (n = 3 mice per group). Statistical analysis was performed using one-way ANOVA followed by Tukey’s multiple-comparisons test. No significant differences were observed among groups.

Figure S35. Novel object recognition (NOR) test for evaluation of recognition memory after different treatments. (a) Schematic illustration of the NOR test procedure. Mice were first habituated in an empty arena, followed by the training phase with two identical objects. In the testing phase, one of the familiar objects was replaced with a novel object, and the preference for the novel object was assessed. (b) Quantification of the preference index in the indicated groups. The preference index was calculated based on the exploration time of the novel object relative to the total exploration time. Data are presented as mean ± s.d. (n = 3 mice per group). Statistical analysis was performed using one-way ANOVA followed by Tukey’s multiple-comparisons test. No significant differences were observed among groups.

Figure S36. *In vivo* assessment of different treatments. (a) Representative H&E-stained coronal sections of mouse brains following treatment with PBS, IVT, or ES. The tumor regions are outlined (black circles), and higher-magnification insets provide detailed views of the tissue architecture. Scale bars: 2 mm (main images), 50 µm (insets). (b) Body weight changes in mice over time for each treatment group, illustrating the systemic impact of PBS, IVT, or ES (n = 6 mice, mean ± s.d.).

Figure S37. Therapeutic efficacy of eye-brain axis electrical stimulation in intracranial GL261 GBM. ES of the eye-brain axis inhibited tumor growth and prolonged the survival of BALB/c mice bearing intracranial GL261 GBM. (a) Schematic of the schedule of procedures and representative *in vivo* bioluminescence imaging of mice bearing intracranial GL261-luc GBM after different treatments with on days 6, 10, and 15 post tumor inoculation. (b) Semi-quantitative results of tumor burden by bioluminescence intensity (n = 5 mice). (c) Kaplan–Meier survival curve of intracranial GL261 GBM-bearing mice after various treatment (n = 5 mice). Statistical significance was analyzed using one-way ANOVA followed by Tukey’s multiple-comparisons test. Adjusted P values for the indicated comparisons are shown in the figure. **P < 0.05*, ***P < 0.01*, ****P < 0.001*, *****P < 0.0001*.

Figure S38. *In vivo* bioluminescence imaging and survival analysis of intracranial GL261-luc GBM under different treatments. (a) Schematic representation of the experimental procedure timeline outlined below. Representative *in vivo* bioluminescent images of mice bearing intracranial GL261-luc GBM-receiving various treatments on days 6, 10, and 15 post tumor inoculation. (b) Tumor burden quantification 6, 10, and 15 days after tumor inoculation (n = 5 mice). (c) Kaplan–Meier survival curve of intracranial GL261 GBM-bearing mice receiving various treatments (n = 6 mice). Statistical significance was analyzed using one-way ANOVA followed by Tukey’s multiple-comparisons test. Adjusted P values for the indicated comparisons are shown in the figure. **P < 0.05*, ***P < 0.01*, ****P < 0.001*, *****P < 0.0001*.

Figure S39. Orthotopic glioma model in nude mice. (a) Schematic representation of the experimental procedure timeline outlined below. (b) Representative *in vivo* bioluminescent images of mice bearing intracranial GL261-luc GBM-receiving various treatments on days 6, 10, and 16 post tumor inoculation.

Figure S40. Tumor growth quantification and survival analysis in intracranial GL261 GBM mice. (a) Tumor burden quantification 6, 10, and 16 days after tumor inoculation (n = 5 mice). (b) Kaplan–Meier survival curve of intracranial GL261 GBM-bearing mice receiving various treatments (n = 5 mice). Statistical significance was analyzed using one-way ANOVA followed by Tukey’s multiple-comparisons test. Longitudinal tumor-burden data were analyzed using two-way ANOVA followed by Sidak’s multiple-comparisons test. Survival curves were compared using the log-rank test. Adjusted P values for the indicated comparisons are shown in the figure. **P < 0.05*, ***P < 0.01*, ****P < 0.001*, *****P < 0.0001*.

Figure S41. ES alone suppresses tumor progression and attenuates glioma-associated synaptic connectivity in GL261-bearing nude mice. (a) Quantification of intracranial tumor burden based on bioluminescence imaging in GL261-bearing nude mice with or without ES treatment at days 6, 10, and 16 after tumor inoculation. (b) Kaplan–Meier survival curves of GL261-bearing nude mice in the Control and ES groups. (c) Representative immunofluorescence images of Synapsin-1 and NFH/M in tumor regions from Control and ES-treated nude mice. Synapsin-1-positive presynaptic puncta are shown in red, and NFH/M-positive neuronal fibers are shown in white. Scale bars, 20 μm. (d) Quantification of the Synapsin-1/NFH/M puncta ratio in tumor regions. (e) Representative immunofluorescence images of PSD95 and NFH/M in tumor regions from Control and ES-treated nude mice. PSD95-positive postsynaptic puncta are shown in red, and NFH/M-positive neuronal fibers are shown in white. Scale bars, 20 μm. (f) Quantification of the PSD95/NFH/M puncta ratio in tumor regions. Data are presented as mean ± s.d. Statistical significance was analyzed using two-way ANOVA followed by Sidak’s multiple-comparisons test in (a), log-rank test in (b), and unpaired two-tailed Student’s *t*-test in (d, f). NS, not significant; **P < 0.05; **P < 0.01; ***P < 0.001.*

To distinguish the neural contribution of ES from adaptive immune activation, we evaluated ES alone in GL261-bearing nude mice. ES reduced intracranial tumor burden, prolonged median survival from 24 to 30 days, and decreased Synapsin-1/NFH/M and PSD95/NFH/M puncta ratios in tumor regions, indicating attenuated glioma-associated synaptic connectivity. These findings suggest that ES-mediated neural modulation partially suppresses glioma progression independently of a fully competent adaptive immune system, while the stronger efficacy of IVT+ES in immunocompetent mice supports a cooperative immune–neural mechanism.

# Supplementary Tables

## Table S1. *In vivo* frequency-screening experiment used to select the working ES condition for subsequent studies.

| Group | Frequency | Duration | Treatment schedule | Readout time point | Relative luminescence intensity at Day 10 | Statistical significance (vs. control) |
| --- | --- | --- | --- | --- | --- | --- |
| Control | — | — | — | Day 10 | 12.16 ± 2.41 | — |
| ES (50 kHz) | 50 kHz | 5 min/session | 5 consecutive days | Day 10 | 10.55 ± 2.07 | NS |
| ES (200 kHz) | 200 kHz | 5 min/session | 5 consecutive days | Day 10 | 6.92 ± 0.67 | ****P < 0.001* |
| ES (300 kHz) | 300 kHz | 5 min/session | 5 consecutive days | Day 10 | 7.32 ± 1.38 | ***P < 0.01* |

Data are presented as mean ± s.d. (n = 3 mice per group). Statistical significance was analyzed using one-way ANOVA followed by Dunnett’s multiple-comparisons test versus the control group. NS, not significant. ***P < 0.01, ***P < 0.001.*

## Table S2. The antibodies used in this study.

| Antibodies | Manufacturer | Catalog  Number | Dilutions | Clone  Numbers | Lot  number |
| --- | --- | --- | --- | --- | --- |
| FITC anti-mouse  CD3 | Biolegend | 100203 | 1:200 | 17A2 | B388790 |
| FITC anti-mouse  F4/80 | Biolegend | 123108 | 1:200 | BM8 | B407719 |
| APC anti-mouse CD4 | Biolegend | 100411 | 1:200 | GK1.5 | B372225 |
| APC anti-mouse CD80 | Biolegend | 104714 | 1:200 | 16-10A1 | B381699 |
| APC anti-mouse CD206 | Biolegend | 141708 | 1:200 | C068C2 | B385870 |
| PE anti-mouse CD8a | eBioscience | 12-0081-82 | 1:200 | 53-6.7 | 2555841 |
| PE anti-mouse CD11b | Biolegend | 101208 | 1:200 | M1/70 | B400893 |

| Antibodies | Manufacturer | Catalog  Number | Dilutions | Lot  number |
| --- | --- | --- | --- | --- |
| HOME1 Polyclonal Antibody | UpingBio | YP-Ab-05672 | 1:200 | 241011 |
| Synapsin I Polyclonal Antibody | UpingBio | YP-Ab-00739 | 1:200 | 240906 |
| PSD-95 Polyclonal Antibody | UpingBio | YP-Ab-12796 | 1:200 | 241011 |
| Goat anti-Rabbit DyLight 649 | UpingBio | FD0130 | 1:200 | 20240807 |
| Goat-anti-Guinea pig IgG(H+L), 488nm | Oasis Biofarm | G-GP488 | 1:1000 | 20240909 |
| MAP2 Guinea pig pAb | Oasis Biofarm | OB-PGP079 | 1:500 | N230522C2-6 |

# Reference

1. D. Kim, B. Langmead, and S. L. Salzberg, “HISAT: A Fast Spliced Aligner with Low Memory Requirements,” *Nature Methods* 12 (2015): 357-360.
2. A. Dobin, C. A. Davis, F. Schlesinger, et al., “STAR: Ultrafast Universal RNA-Seq Aligner,” *Bioinformatics* 29 (2013): 15-21.
3. S. K. Tetzlaff, E. Reyhan, N. Layer, et al., “Characterizing and Targeting Glioblastoma Neuron-Tumor Networks with Retrograde Tracing,” *Cell* 188 (2025): 390–411.
4. Y. Zhang, W. Duan, L. Chen, et al., “Potassium Ion Channel Modulation at Cancer-Neural Interface Enhances Neuronal Excitability in Epileptogenic Glioblastoma Multiforme,” *Neuron* 113 (2025): 225–243.
5. P. Fluxá, D. Rojas-Sepúlveda, M. A. Gleisner, et al., “High CD8^+^ and Absence of Foxp3^+^ T Lymphocytes Infiltration in Gallbladder Tumors Correlate with Prolonged Patients’ Survival,” *BMC Cancer* 18 (2018): 243.
6. Y. Huang, C. Ma, Q. Zhang, et al., “CD4^+^ and CD8^+^ T Cells Have Opposing Roles in Breast Cancer Progression and Outcome,” *Oncotarget* 6 (2015): 17462–17478.
7. E. M. Janssen, E. E. Lemmens, T. Wolfe, et al., “CD4^+^ T Cells Are Required for Secondary Expansion and Memory in CD8^+^ T Lymphocytes,” *Nature* 421 (2003): 852–856.
8. D. J. Shedlock and H. Shen, “Requirement for CD4 T Cell Help in Generating Functional CD8 T Cell Memory,” *Science* 300 (2003): 337–339.
